# Supplementary material for: The Structure of Clostridioides difficile SecA2 ATPase Exposes Regions Responsible for Differential Target Recognition of the SecA1 and SecA2-Dependent Systems
Source: Int J Mol Sci. 2020 Aug 26;21(17):6153. doi: 10.3390/ijms21176153 (PMC7503281; doi:10.3390/ijms21176153)
Supplement: Supplementary file 1 [file ijms-21-06153-s001.pdf]

SUPPLEMENTAL MATERIAL:

The structure of *Clostridioides difficile* SecA2 ATPase exposes regions responsible for differential target recognition of the SecA1 and SecA2-dependent systems

Nataša Lindič [1], Jure Loboda [1], Aleksandra Usenik [1,2], Robert Vidmar [1] and Dušan Turk [1,2*]

[1] Department of Biochemistry, Molecular and Structural Biology, Jozef Stefan Institute, Jamova cesta 39, 1000 Ljubljana, Slovenia

[2] Centre of Excellence for Integrated Approaches in Chemistry and Biology of Proteins (CIPKeBiP), Jamova cesta 39, 1000 Ljubljana, Slovenia

* Correspondence: dusan.turk@ijs.si; Tel.: +386 1 477 3857

SUPPLEMENTAL FIGURES:

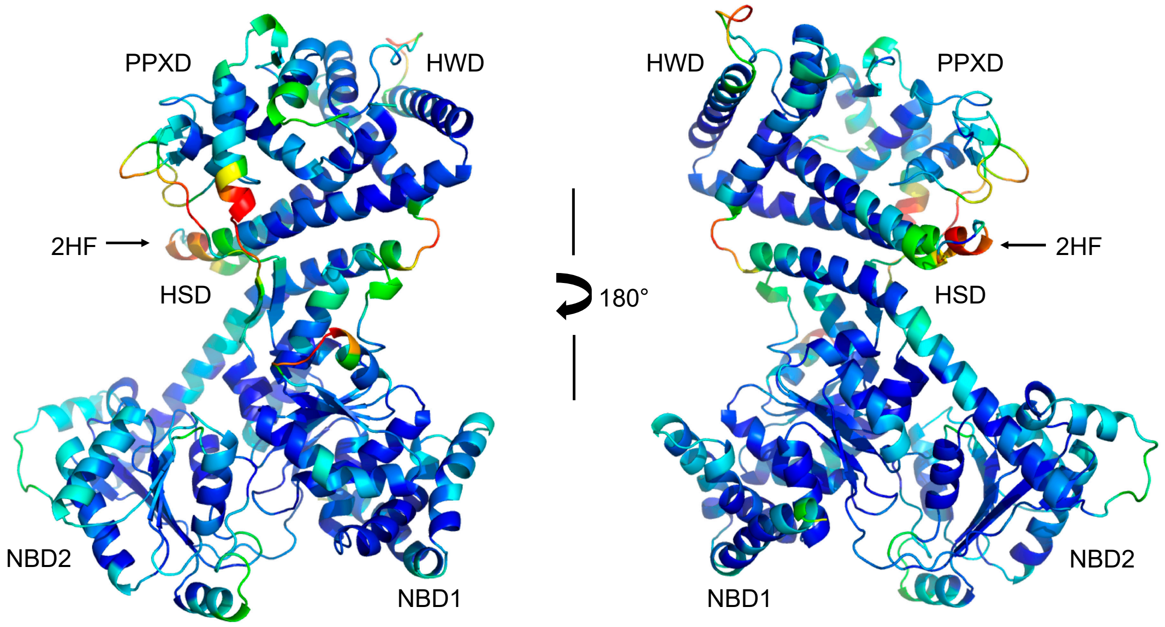

Figure S1, related to Figure 1: B-factor representation of the CDSecA2 crystal structure. All the objects are coloured according to the B-factor values (blue, low B-factors; red, high B-factors), and domains are depicted. 2HF with its loop is pointed out with an arrow. The molecule is shown in two different orientations. The figure was made with PyMOL [1].

```
Conservation:                                                                                         5  5           6  7   5  5
SECA1_GEO_thermodenitrifi    1  ---------------------------------------MLGV--LKKV-------FDPNKRQLARLEKIADQVDALGPEMARLSDEQLRQKTEEFKARY-------QQGES--------   57
SECA1_GEO_kaustophilus       1  ---------------------------------------MLGV--LKKV-------FDPNKRQLARLEKIADQVDALGPEMARLSDEQLRQKTEEFKARY-------QQGES--------   57
SECA1_LAC_johnsonii          1  ---------------------------------------MANI--LKKI-------YDNDRRELKKFEKLATKVESLGDEYEKLSDEQLQAKTPEFRKRL-------KNGET--------   57
SECA1_STR_parasanguinis      1  ---------------------------------------MANL--LKTI-------IENDRGEIKRLEKMADKVFSYEDQMAALTDDELKAKTVEFKQRY-------QDGES--------   57
SECA1_STR_gordonii           1  ---------------------------------------MANI--LRTI-------IENDKGELRKLEKMANKVIAYSDQMAALSDEELKAKTDEFKQRY-------QNGES--------   57
SECA1_STR_pneumoniae         1  ---------------------------------------MANI--LKTI-------IENDKGEIRRLEKMADKVFKYEDQMAALTDDQLKAKTVEFKERY-------QNGES--------   57
SECA1_STR_agalactiae         1  ---------------------------------------MANI--LRTV-------IENDKGELKKLDKIAKKVDSYADHMAALSDEALQAKTPEFKERY-------QNGET--------   57
SECA1_LIS_monocytogenes      1  ---------------------------------------MAGL--LKKI-------FESGKKDVKYLERKADEIIALADETAALSDDALREKTVEFKERV-------QKGET--------   57
SECA1_LIS_innocua            1  ---------------------------------------MAGL--LKKI-------FESGKKDVKYLERKADEIIALADETAALSDEALREKTVEFKERV-------QKGET--------   57
SECA1_LIS_welshimeri_        1  ---------------------------------------MAGL--LKKI-------FESGKKDVKYLERKADEIIALADETAALSDDALREKTVEFKERV-------QKGET--------   57
SECA_BAC_subtilis            1  ---------------------------------------MLGI--LNKM-------FDPTKRTLNRYEKIANDIDAIRGDYENLSDDALKHKTIEFKERL-------EKGAT--------   57
SECA1_BAC_anthracis          1  ---------------------------------------MIGI--LKKV-------FDVNQRQIKRMQKTVEQIDALESSIKPLTDEQLKGKTLEFKERL-------TKGET--------   57
SECA1_BAC_thuringiensis      1  ---------------------------------------MIGI--LKKV-------FDVNQRQIKRMQKTVEQIDALESSIKPLTDEQLKGKTLEFKERL-------TKGET--------   57
SECA1_BAC_cereus             1  ---------------------------------------MIGI--LKKV-------FDVNQRQIKRMQKTVEQIDALESSIKPLTDEQLKGKTLEFKERL-------TKGET--------   57
SECA1_STA_epidermidis_       1  ----------------------------------------MGF--LSKI-------VDGNKKEIKRLGKLADKVLALEEDTAILTDEEIREKTKSFQKELAEIEDVKKQNDY--------   63
SECA1_STA_haemolyticus       1  ----------------------------------------MGF--LSKI-------VDGNKKETKRLSKIADEVLSLEEDMAILTDEEIKNKTKQFQQEVQEIEDVKKQNDY--------   63
SECA1_STA_aureus             1  ----------------------------------------MGF--LSKI-------LDGNNKEIKQLGKLADKVIALEEKTAILTDEEIRNKTKQFQTELADIDNVKKQNDY--------   63
SECA1_ALK_metalliredigens    1  ---------------------------------------MKRL--FEKV------FGSESEREIKKIDKLADRVEALDEEYKKLSDQALQSKTAELKGRL-------SQGEA--------   58
SECA1_PEP_difficile          1  ----------------------------------------MSF--MDNL------FNMADKKELKKFNKTVDIIDSLEPKFESMADSELKNMTNIFKERL-------ANGES--------   57
SECA2_PEP_difficile          1  ----------------------------------------MSV--IDSI------LDKADEQEIKKLNVIVDKIDALEDSMKNLSYEELKDMTAIFKNRL-------KKGET--------   57
SECA2_model_opened_conf      1  ------------------------------------------------------------EQEIKKLNVIVDKIDALEDSMKNLSYEELKDMTAIFKNRL-------KKGET--------   45
SECA_E_coli_                 1  --------------------------------------MLIKL--LTKV------FGSRNDRTLRRMRKVVNIINAMEPEMEKLSDEELKGKTAEFRARL-------EKGEV--------   59
SECA1_MYC_vanbaalenii        1  ------------------------------------------M--LSKL------LRLGEGRMVKRLKGVADYVNTLSDDIEKLSDAELRGKTDEFRARL-------AGGKE--------   55
SECA1_MYC_ulcerans           1  ------------------------------------------M--LSKL------LRVGEGRMVKRLKKVADYVESLSGDVEKLTDAELRAKTDEFKKRH-------AEGES--------   55
SECA1_MYC_paratuberculosi    1  ------------------------------------------M--LSKL------LRLGEGRMLKRLKRVADYVNTLSDEVEKLTDAELRAKTDEFKKRH-------ADGES--------   55
SECA1_MYC_tuberculosis       1  ------------------------------------------M--LSKL------LRLGEGRMVKRLKKVADYVGTLSDDVEKLTDAELRAKTDEFKRRLA----DQKNPET--------   58
SECA1_MYC_bovis              1  ------------------------------------------M--LSKL------LRLGEGRMVKRLKKVADYVGTLSDDVEKLTDAELRAKTDEFKRRLA----DQKNPET--------   58
SECA1_MYC_leprae             1  ------------------------------------------M--LSKL------LRLGEGRIVKRLKRVAEYVNTLSDNVEKLTDVELKAKTDEFKQRLA----DEKNPES--------   58
SECA1_MYC_smegmatis          1  ------------------------------------------M--LSKL------LRLGEGRMVKRLRKVADYVNALSDDVEKLSDAELRAKTEEFKKRV-------ADGED--------   55
SECA1_COR_diphtheriae        1  ------------------------------------------MFGLSKM------LRVGEGRAVKRLKKIADDVIALEADYTDLTDEELKAKTHEFQERI-------AQGES--------   57
SECA1_COR_efficiens          1  ------------------------------------------MFGLSKM------LRVGEGRAVKRLQKIADQVIALEDQYANLTDEELKAKTEEFKDRL-------AQGET--------   57
SECA1_COR_glutamicum         1  ------------------------------------------MFGLSKV------LRVGEGRAVKRLHKIADQVIALEDKFANLTDEELKAKTAEFKERI-------AGGEG--------   57
SECA1_COR_jeikeium           1  ------------------------------------------MLGLSKI------LRMGEGRAVKRLAKIADQVMDLDEEYTKLTDEELQAKTDELKKRV------QEDGES--------   58
SECA1_THE_fusca              1  -----------------------------------------MPGILDRV------LRIGEGKILRKLNKLKDQINSIEDDFVDLSDAELRALTDEYKQRL-------KDGEE--------   58
SECA2_MYC_vanbaalenii        1  ------------------------------------------M--LADV------LRLGEARTVKQLSAIADHVDSLARGVENLTDAELSSRTDVFRRRV-------ADGDV--------   55
SECA_THE_maritima_           1  -----------------------------------------------MI------LFDKNKRILKKYAKMVSKINQIESDLRSKKNSELIRLSMVLKEKV-------NSFED--------   52
SECA2_GEO_kaustophilus       1  ---------------------------------------MFAK--VKQL-------FDESAREVQRLAKLAAQINEWEPTISSLSDEQLRQKTVVFKERL-------ERGET--------   57
SECA2_GEO_thermodenitrifi    1  ---------------------------------------MLSL--LKRA------IGYTNERQLKKYMRVVEQINRMEPQMEKLTDAELRRKTDEFKEQL-------ASGKS--------   58
SECA2_BAC_anthracis          1  ---------------------------------------MLNS--VKKL------LGDSQKRKLKKYEQLVQEINNLEEKLSDLSDEELRHKTITFKDML-------RDGKT--------   58
SECA2_BAC_thuringiensis      1  ---------------------------------------MLNS--VKKL------LGDSQKRKLKKYEQLVQEINNLEEKLSDLSDEELRHKTITFKDML-------RDGKT--------   58
SECA2_BAC_cereus             1  ---------------------------------------MLNS--VKKL------LGDSQKRKLKKYEQLVQEINNLEEKLSDLSDEELRHKTITFKDML-------RDGKT--------   58
SECA2_LIS_welshimeri         1  ---------------------------------------------MKQN--------FDDRKIVKQYREIARQIVKKEGLYKNMDQDELREQTNYWREKF-------KTKEM--------   52
SECA2_LIS_monocytogenes      1  ---------------------------------------------MRQN--------YDDRKIVKQYREIARQIVKKEGLYKNMEQAELCEQTNFWREKF-------KTKPM--------   52
SECA2_LIS_innocua            1  ---------------------------------------------MRQN--------YDDRKIVKQYREIARQIVRKEGLYKNMDQDELREQTNFWREKF-------KTKPM--------   52
SECA2_ALK_metalliredigens    1  ---------------------------------------MLKN--ISSVSKRFENFKNKEVYDIELYKKTVEEINNI--NYKNASDTELREISENLKNKV-------KEGIP--------   62
SECA2_LAC_johnsonii          1  --------------------------------------------------------MLTDRLRLKKARKLLNKINKLGPKMQAMSDEKLQGQTAIFKKQL-------KEGKS--------   49
SECA2_STA_haemolyticus       1  ------------------------------------------M--ANQV------SNVINSMRLKRLQKQLVAVNRLSDQMRNCSDEALQAKTADFKQRL-------EKRET--------   55
SECA2_STA_aureus             1  ------------------------------------------M--KHKL------DVTINELRLKSIRKIVKRINTWSDEVKSYSDDALKQKTIEFKERL-------ASGVD--------   55
SECA2_STA_epidermidis        1  ------------------------------------------M--AKGV------NQIINNIRLRKLRKILNQINALSEEFSNFSDEALQAKTKEFKVYL-------NDNKA--------   55
SECA2_STR_Agal               1  ----------------------------------------MTA--FNSL-------FSLDKKRLKKLQRTLNTINSLKDQMATLSNEELQAKTTEFRKRL-------VNGET--------   56
SECA2_STR_Gordon             1  ------------------------------------------M--VKNF------------FHIRRLKKILAKIKSFEKEMSSLSDLDLQKKTDEFKKRL-------ADGEN--------   49
SECA2_STR_Pneum              1  -----------------------------------------MFRRLGQD------------FQLRKVKKILKQINALKGKMSSLSDQELVAKTVEFRQRL-------SEGES--------   52
SECA2_STR_Sanguin            1  ------------------------------------------M--IKNH------------FQIQRLKKILAKVKSFESEMAGLTDAELRKKTQEFKERL-------AAGET--------   49
SECA2_COR_efficiens          1  ---------------------------------------MAGFDWFWKA------LGGKQGRNQKRSLAIVDQAEAHTRDLEALDDAHLAARA---RTLV-------GDG----------   55
SECA2_MYC_ulcerans           1  --MRSCTAAPGVSRTNTHARRGLGVAYRWAVSKTTRAQSGRLSSRFWRL------LGATTEKNQNRSLAQVTASAEFDKEAADLNDEKLRKAA---GLLN-------LEDLA--------   94
SECA2_MYC_paratuberculosi    1  ------------------------------MPKTNRAQPGRLSSRFWRL------LGASTEKNRSRSLTLVTDSSEYDDEAAGLTDEQLRKAA---GLLN-------LEDLA--------   66
SECA2_MYC_tuberculosis       1  MNVHGCPRIAACRCTDTHPRGRPAFAYRWFVPKTTRAQPGRLSSRFWRL------LGASTEKNRSRSLADVTASAEYDKEAADLSDEKLRKAA---GLLN-------LDDLA--------   96
SECA2_MYC_bovis              1  MNVHGCPRIAACRCTDTHPRGRPAFAYRWFVPKTTRAQPGRLSSRFWRL------LGASTEKNRSRSLADVTASAEYDKEAADLSDEKLRKAA---GLLN-------LDDLA--------   96
SECA2_MYC_leprae             1  ------------------------------MSKTPRTQPGRLSSRFWRL------LGASTDKNLNYSSAEVTAAAAYHKEAADLGDEQLRKAC---GLLN-------LNDLA--------   66
SECA2_MYC_smegmatis          1  ------------------------------MPKTSSAKPGRLSSKFWKL------LGASTERNQARSLSEVKGAADFEKKAADLDDEQLTKAA---KLLK-------LEDLA--------   66
SECA2_THE_fusca              1  ---------------------------------------MAEG--VRRL------LGKPGSVSLQPYIKLLKTIEEREEALRKLSDAELTEVA---TELG-------NAELP--------   55
SECA2_COR_diphtheriae        1  ---------------------------------------MAGFRWFWDA------MGGKNTRNQTKSKNIVAQAAKRGVQFASLSDADVVARA---HECA-------QHS----------   55
SECA2_COR_glutamicum         1  ---------------------------------------MAGFDWFWKA------LGGKSGRNQKRSVAIVNQVENHAAELDALDDVALAQRA---KDLA-------SGG----------   55
SECA2_COR_jeikeium           1  ---------------------------------------MAFDW-FWKA------MGSSPKKNQKKSRAVVAQAD--SSRYSGLSDAELRDAA---SDVV-------TEQSTSEDGHHFG   62
Consensus_aa:                   .............................................h.ph..........p..plpph.phhppl..hp.ph..hoD.pLp..o..F+p.h........psbs.........
Consensus_ss:                                                             h  hhhh         hhhhhhhhhhhhhhhhhhhhhhhh   hhhhhhhhhhhhhhh       h
```

Walker A

```
Conservation:                         5585 6765 9 565 55 89  7   6   8 6 89 9999999    6 55 66 9 565568 9 996 78   6  5   685
SECA1_GEO_thermodenitrifi   58  -LDDLLVEAFAVVREGAKRVLGLYPYKVQIMGGVVLHEGDIAEMKTGEGKTLTATMPVYLNALTGRGVHVVTVNEYLASRDAKEMGQLYEFLGLTVGLNLSGM-----------------  159
SECA1_GEO_kaustophilus      58  -LDDLLVEAFAVVREGAKRVLGLYPYKVQIMGGVVLHEGDIAEMKTGEGKTLTATMPVYLNALTGRGVHVVTVNEYLATRDATEMGKLYEFLGMTVGLNLSGM-----------------  159
SECA1_LAC_johnsonii         58  -LDDILPEAFATAREGAKRVLGLYPFRVQIIGGIALHYGNIAEMMTGEGKTLTATLPVYLNALTGKGVHVVTVNEYLSSRDESEMGQLYKWLGLSVGLNLNSM-----------------  159
SECA1_STR_parasanguinis     58  -LDDLLYEAFAVVREGAKRVLGLFPYKVQVMGGIVLHHGDVPEMRTGEGKTLTATMPVYLNALSGKGVHVVTVNEYLTERDATEMGELYSWLGLSVGINLAAK-----------------  159
SECA1_STR_gordonii          58  -LDDLLYEAFAVVREGAKRVLGLYPYPVQIMGGIVLHHGDVPEMRTGEGKTLTATMPVYLNALAGEGVHVVTVNEYLTERDATEMGELYSWLGLSVGINLAAK-----------------  159
SECA1_STR_pneumoniae        58  -LDSLLYEAFAVVREGAKRVLGLFPYKVQVMGGIVLHHGDVPEMRTGEGKTLTATMPVYLNALSGKGVHVVTVNEYLSERDATEMGELYSWLGLSVGINLATK-----------------  159
SECA1_STR_agalactiae        58  -LDQLLPEAFAVVREASKRVLGLYPYHVQIMGGIVLHHGDIPEMRTGEGKTLTATMPVYLNAISGLGVHVITVNEYLSTRDATEMGEVYSWLGLSVGINLAAK-----------------  159
SECA1_LIS_monocytogenes     58  -LDDLLVEAFAVAREGAKRALGLYPFKVQLMGGIVLHEGNIAEMKTGEGKTLTATLPVYLNALSGEGVHVVTVNEYLAHRDAEEMGVLYNFLGLSVGLNLNAL-----------------  159
SECA1_LIS_innocua           58  -LDDLLVEAFAVAREGAKRALGLYPFKVQLMGGIVLHEGNIAEMKTGEGKTLTATLPVYLNALSGEGVHVVTVNEYLAHRDAEEMGVLYNFLGLSVGLNLNAL-----------------  159
SECA1_LIS_welshimeri_       58  -LDDLLVEAFAVAREGAKRALGLYPFKVQLMGGIVLHEGNIAEMKTGEGKTLTATLPVYLNALSGEGVHVVTVNEYLAHRDAEEMGVLYNFLGLSVGLNLNAL-----------------  159
SECA_BAC_subtilis           58  -TDDLLVEAFAVVREASRRVTGMFPFKVQLMGGVALHDGNIAEMKTGEGKTLTSTLPVYLNALTGKGVHVVTVNEYLASRDAEQMGKIFEFLGLTVGLNLNSM-----------------  159
SECA1_BAC_anthracis         58  -VDDLLPEAFAVVREAATRVLGMRPYGVQLMGGIALHEGNISEMKTGEGKTLTSTLPVYLNALTGKGVHVVTVNEYLAQRDANEMGQLHEFLGLTVGINLNSM-----------------  159
SECA1_BAC_thuringiensis     58  -VDDLLPEAFAVVREAATRVLGMRPYGVQLMGGIALHEGNISEMKTGEGKTLTSTLPVYLNALTGKGVHVVTVNEYLAQRDASEMGQLHEFLGLTVGINLNSM-----------------  159
SECA1_BAC_cereus            58  -VDDLLPEAFAVVREAATRVLGMRPYGVQLMGGIALHEGNISEMKTGEGKTLTSTLPVYLNALTGKGVHVVTVNEYLAQRDASEMGQLHEFLGLTVGINLNSM-----------------  159
SECA1_STA_epidermidis_      64  -LDKILPEAYALVREGSKRVFNMIPYKVQVMGGIAIHKGDIAEMRTGEGKTLTATMPTYLNALAGRGVHVITVNEYLSSSQSEEMAELYNYLGLTVGLNLNSK-----------------  165
SECA1_STA_haemolyticus      64  -LDKILPQAYALVREGAKRVFNMTPYKVQVMGGIAIHKGDIAEMRTGEGKTLTATMPTYLNALAGRGVHVITVNEYLSSVQSEEMAELYEFLGLTVGLNLNSK-----------------  165
SECA1_STA_aureus            64  -LDKILPEAYALVREGSKRVFNMTPYKVQIMGGIAIHKGDIAEMRTGEGKTLTATMPTYLNALAGRGVHVITVNEYLSSVQSEEMAELYNFLGLTVGLNLNSK-----------------  165
SECA1_ALK_metalliredigens   59  -LDDILPEAFATMREAAWRVLGMKHYRVQIYGAIILHQGRISEMKTGEGKTLMATLPVYLNALAGKGVHVVTVNDYLAQRDCEWMGKLYEFLGLSVGVIVHGI-----------------  160
SECA1_PEP_difficile         58  -IDDILPEAFAVVREVSKRVLGLRHYRVQMIGGIVLHQGRIAEMKTGEGKTLVATAPVYLNALTGKGVHVVTVNDYLAKRDRDQMAKIYEFLGMSVGVIIHGQ-----------------  159
SECA2_PEP_difficile         58  -LDDILPEAFAVVREVSKRKLGMRQYRVQLIGGIVIHQGKIAEMKTGEGKTLVEVAPVYLNALTGKGVHVITVNDYLAERDKELMSPVYESLGMTVGVIISNQ-----------------  159
SECA2_model_opened_conf     46  -LDDILPEAFAVVREVSKRKLGMRQYRVQLIGGIVIHQGKIAEMKTGEGKTLVEVAPVYLNALTGKGVHVITVNDYLAERDKELMSPVYESLGMTVGVIISNQ-----------------  147
SECA_E_coli_                60  -LENLIPEAFAVVREASKRVFGMRHFDVQLLGGMVLNERCIAEMRTGEGKTLTATLPAYLNALTGKGVHVVTVNDYLAQRDAENNRPLFEFLGLTVGINLPGM-----------------  161
SECA1_MYC_vanbaalenii       56  DLDDVMPEAFAVVREAAWRVLNQRHFDVQIMGGAALHFGNVAEMKTGEGKTLTSVLPAYLNALPGKGVHIVTVNEYLAKRDAEQMGRVHRFLGLDVDVILGTL-----------------  158
SECA1_MYC_ulcerans          56  -LDELLPEAFAVAREAAWRVLGQRPFEVQLMGAAALHLGNVAEMKTGEGKTLTSVLPAYLNGIGGKGVHIVTVNDYLAKRDSEWMGRVHRFLGLDVGVILAQM-----------------  157
SECA1_MYC_paratuberculosi   56  -LDDLLPEAFAVAREAAWRVLDQRPFDVQVMGAAALHFGNVAEMKTGEGKTLTSVLPAYLNGIGGKGVHVVTVNDYLAKRDSEWMGRVHRFLGLDVGVILAQM-----------------  157
SECA1_MYC_tuberculosis      59  -LDDLLPEAFAVAREAAWRVLDQRPFDVQVMGAAALHLGNVAEMKTGEGKTLTCVLPAYLNALAGNGVHIVTVNDYLAKRDSEWMGRVHRFLGLQVGVILATM-----------------  160
SECA1_MYC_bovis             59  -LDDLLPEAFAVAREAAWRVLDQRPFDVQVMGAAALHLGNVAEMKTGEGKTLTCVLPAYLNALAGNGVHIVTVNDYLAKRDSEWMGRVHRFLGLQVGVILATM-----------------  160
SECA1_MYC_leprae            59  -LDDLLPEAFAVAREAAWRVLDQRPFDVQVMGGAALHLGNVAEMKTGEGKTLTCVLPAYLNALAGKGVHIVTVNDYLAKRDSEWMGRVHRFLGLQVGVILASM-----------------  160
SECA1_MYC_smegmatis         56  -LDDLLPEAFAVAREAAWRVLNQRHFDVQVMGGAALHFGNVAEMKTGEGKTLTAVLPSYLNALSGKGVHVVTVNDYLARRDSEWMGRVHRFLGLDVGVILSGM-----------------  157
SECA1_COR_diphtheriae       58  -VDDLLLEAFAVAREASWRVLGQKHYPVQIMGGAALHFGNVAEMRTGEGKTLTCVLPAYLNALEGKGVHVVTVNDYLAKRDAEWMGRVHRWLGLNVGVILANM-----------------  159
SECA1_COR_efficiens         58  -LDDIFLDAFATAREASWRVLGQKHYKVQIMGGAALHFGNVAEMRTGEGKTLTCVLPAYLNALEGKGVHVVTVNDYLAKRDAEWMGRVHRWLGLSVGVILSEL-----------------  159
SECA1_COR_glutamicum        58  -LDEIFLEAFATAREAAWRVLGQKHYHVQIMGGAALHFGNVAEMRTGEGKTLTCVLPAYLNALEGKGVHVVTVNDYLAKRDAEMMGRVHRYLGLEVGVILSDM-----------------  159
SECA1_COR_jeikeium          59  -LDDILLEAFATAREASWRVLGQKHYKVQIMGGAGLHFGYVSEMKTGEGKTLTCVLPAYLNALSGKGVHVVTVNDYLAKRDAEWMGRVHRFLGLSTDVILSGK-----------------  160
SECA1_THE_fusca             59  -LDDLLPEAFATVREAAKRTLGQRHFDVQLMGGAALHFGNIAEMKTGEGKTLTATLPVYLNALTGKGVHVVTVNDYLARRDAETMGRIYRFLGMEVGVISPEM-----------------  160
SECA2_MYC_vanbaalenii       56  -LDELLPEGFAVAREAAWRVLGLRPYHVQVMGGAALHFGNIAEMMTGEGKTLACVMPAYLNAIGGKGVHIVTVNDYLAGRDAEQMGRVHRFLGLTVGVILSEM-----------------  157
SECA_THE_maritima_          53  -ADEHLFEAFALVREAARRTLGMRPFDVQVMGGIALHEGKVAEMKTGEGKTLAATMPIYLNALIGKGVHLVTVNDYLARRDALWMGPVYLFLGLRVGVINSLGKSYEVVWKNPDLARKAI  171
SECA2_GEO_kaustophilus      58  -LDDIKIEAFAVVREAARRVLGLRHYDVQLMGGLAMHEGNIAEMQTGEGKTLAATLPSYLHALLGKGVHIITANEYLARRDCEQMGRVFRFLGLTVGLNVSQM-----------------  159
SECA2_GEO_thermodenitrifi   59  -VNDIQVEAFAVVREVAKRVLGMRHFDVQLIGGLVLAEGNIAEMATGEGKTLVASLPSYLRALEGKGVHVITANDYLAKRDRNLIGQIHEFLGLTVGLNLPLM-----------------  160
SECA2_BAC_anthracis         59  -VDDIKVEAFAVVREAAKRVLGLRHYDVQLIGGLVLLEGNIAEMPTGEGKTLVSSLPTYVRALEGKGVHVITVNDYLAKRDKELIGQVHEFLGLKVGLNIPQI-----------------  160
SECA2_BAC_thuringiensis     59  -VDDIKVEAFAVVREAAKRVLGLRHYDVQLIGGLVLLEGNIAEMPTGEGKTLVSSLPTYVRALEGKGVHVITVNDYLAKRDKELIGQVHEFLGLKVGLNIPQI-----------------  160
SECA2_BAC_cereus            59  -VDDIKVEAFAVVREAAKRVLGLRHYDVQLIGGLVLLEGNIAEMPTGEGKTLVSSLPTYVRALEGKGVHVITVNDYLAKRDKELIGQVHEFLGLKVGLNIPQI-----------------  160
SECA2_LIS_welshimeri        53  -SERDKINIFALAREAASRIIGLDAVVVQLIGALVLGDGKVAEMKTGEGKTLMSLFVMFIEVMRGNRVHLVTANEYLARRDREEIGQVLEYLGISVALNESDL-----------------  154
SECA2_LIS_monocytogenes     53  -TDRDKINIFALAREAASRIIGLDAVVVQLIGALVLGDGKVAEMKTGEGKTLMSLFVMFIEVMRGNRVHLVTANEYLARRDREEIGQVLEYLGVSVALNESGL-----------------  154
SECA2_LIS_innocua           53  -TERDKINIFALAREAASRIIGLDAVVVQLIGALVLGDGKVAEMKTGEGKTLMSLFVMFIEVMRGNRVHLVTANEYLARRDREEIGQVLEYLGISVALNESGL-----------------  154
SECA2_ALK_metalliredigens   63  -LDEVLIQAFTLVKEVAGRVVGMTPYDVQKMAAIALHQGKVVEMQTGEGKTLSAVMPAYLNALSGKGVHILTFNDYLAERDFRWMGPIYKFLGLSCGFVKENM-----------------  164
SECA2_LAC_johnsonii         50  -LDDILPEAYATVREADKRILGMFPYDVQVLGAIVLHNGSIAEMKTGEGKTLVATMALYLNALEGKGAMLVTPNGYLASRDKKELAPVYEWLGLSVSLAFAEEKDS--------------  154
SECA2_STA_haemolyticus      56  TLDKLLPEAYATIREASKRVLGMYPKDVQVMGAIVMHQGNIAEMQTGEGKTLTATMPLYLNALTGKSAFLITTNDYLANRDFQEMRPLYEWLGLTASLGFVDIPD---------------  160
SECA2_STA_aureus            56  TLDTLLPEAYAVAREASWRVLGMYPKEVQLIGAIVLHEGNIAEMQTGEGKTLTATMPLYLNALSGKGTYLITTNDYLAKRDFEEMQPLYEWLGLTASLGFVDIVD---------------  160
SECA2_STA_epidermidis       56  SLNHILPQAYATVREASKRVLGMYPKDVQILGAIAMHQGNIAEMQTGEGKTLTATMPLYLNALTGKGAYLITTNDYLAKRDFLEMKPLYEWLGLSVSLGFVDIPE---------------  160
SECA2_STR_Agal              57  -LDDICAEAFAVVREADKRVLGLFPYDVQVIGGLVLHQGNTAEMKTGEGKTLTATMPLYLNALEGKGAMLLTNNSYLAIRDAEEMGKVYRFLGLSVGVGVSDNEE---------------  160
SECA2_STR_Gordon            50  -LDQLLPEAYAVVREVDKRILGLFPYDVQVMGAIVLHEGNIAEMATGEGKTLTATMPLYLNALSGQGAMLVTPNSYLALRDAKEMGPVYRFLGLTIETAVRADES---------------  153
SECA2_STR_Pneum             53  -LDDILVEAFAVVREADKRILGMFPYDVQVMGAIVMHYGNVAEMNTGEGKTLTATMPVYLNAFSGEGVMVVTPNEYLSKRDAEEMGQVYRFLGLTIGVPFTEDPK---------------  156
SECA2_STR_Sanguin           50  -LDDLLPEAYAVVREADKRVLGMFPYDVQVMGAIVLHEGNVAEMATGEGKTLTATMPLYLNALSGQGAMLVTTNTYLALRDAQEMGQVYRFLGLTIEAAVVADET---------------  153
SECA2_COR_efficiens         56  -TPQDPAELLAVLGIAAHRTLGMRPFPVQSQAVLRLIEGDVVHMATGEGKTLVGAMAATGLGLQGKTVHSITINDYLAVRDAEWMRPLVEFFGLSVGAISETM-----------------  157
SECA2_MYC_ulcerans          95  -DSADIPQFLAIAREAGERATGLRPFDVQLLGALRMLAGDVIEMATGEGKTLAGAIAAAGYALGGRHVHVVTINDYLARRDAEWMAPLLEAMDLTVGWITAES-----------------  196
SECA2_MYC_paratuberculosi   67  -ESEDIPQFLAIAREAAERATGLRPFDVQLLGALRMLAGDVIEMATGEGKTLAGAIAAAGYVLAGRHVHVVTINDYLARRDAEWMGPLIEAMGLTVGWITAES-----------------  168
SECA2_MYC_tuberculosis      97  -ESADIPQFLAIAREAAERRTGLRPFDVQLLGALRMLAGDVIEMATGEGKTLAGAIAAAGYALAGRHVHVVTINDYLARRDAEWMGPLLDAMGLTVGWITADS-----------------  198
SECA2_MYC_bovis             97  -ESADIPQFLAIAREAAERRTGLRPFDVQLLGALRMLAGDVIEMATGEGKTLAGAIAAAGYALAGRHVHVVTINDYLARRDAEWMGPLLDAMGLTVGWITADS-----------------  198
SECA2_MYC_leprae            67  -DSRDVPQFLAIVREASERSTGLRPFDVQLLGALRMLAGDVIEMATGEGKTLAGAIAAAGYALAGRHVHVVTINDYLARRDAEWMGPLLEAIGLTVGWITAES-----------------  168
SECA2_MYC_smegmatis         67  -GASDITQFLAIAREAAERTTGLRPFDVQLLAALRMLAGDVVEMATGEGKTLAGAIAAAGYALGGRRVHVITINDYLARRDAEWMGPLLKALGLTVGWITADS-----------------  168
SECA2_THE_fusca             56  YDRDDLAELCALGREAARRTLGERPFDTQLIGMMALLDGHVAEMATGEGKTLTGALAAVGFALRGQRVHVLSVNDYLARRDAEWMRPLYTLLGVEVGWISQES-----------------  158
SECA2_COR_diphtheriae       56  -DDESRADLLALLSIGAQRSLSMNPFNVQLQAVLRILEGDVIHMATGEGKTLVGAMASVGYALQGKRVHSITVNDYLAERDAEWMGDLVRYFGLTVSAVTESL-----------------  157
SECA2_COR_glutamicum        56  -RIDNHAEFLAILGVASQRTLGLKPYPVQSQAVLRLIEGDVVHMATGEGKTLVGAMAATGLGLMGKRVHSITVNDYLAVRDAEWMRPLVEFFGLSVASISEKM-----------------  157
SECA2_COR_jeikeium          63  GQVDDAPALLAILREAASRTLNMEPFDVQMQGTYRLLHGDVVEMATGEGKTLAGAMAAVGFALQGKRVHVITVNSYLAGRDNDWMGPMFDFFGLTHGAIREDL-----------------  165
Consensus_aa:                   .hcc.h.ph@AlhREtt.RhlGh..@cVQlhGthhlh.GplhEM.TGEGKTLhthhsh@lpAL.Gc.V@llThN-YLApRD.pbMt.lhc@LGLoVth.....................
Consensus_ss:                    hhhhhhhhhhhhhhhhhhhh      hhhhhhhhhhh   eeee      hhhhhhhhhhhhhh     eeeee   hhhhhhhhhhhhhhhhhh   eeeee
```

```
                                                                                                               Walker B
Conservation:                                                                     75   9  7768 5  5 8997957575      6     66669969677695956976676                   5
SECA1_GEO_thermodenitrifi 160   ---------------------------------SREEKQAAYNADITYGTNNEFGFDYLRDNMVLYKEHIVQRPLYFAVVDEVDSILIDEARTPLIISGT-AQKSTKLYVQANAFVRTLR   245
SECA1_GEO_kaustophilus    160   ---------------------------------SREEKQAAYNADITYGTNNEFGFDYLRDNMVLYKEHIVQRPLYYAIIDEVDSILIDEARTPLIISGT-AQKSTKLYVQANAFVRTLR   245
SECA1_LAC_johnsonii       160   ---------------------------------SADEKRDAYNCDVTYSTNSELGFDYLRDNMVVYKDQMVQRPLNYAIIDEVDSILIDEARTPLIISGQ-AEQANSEYIRADRFVKTLV   245
SECA1_STR_parasanguinis   160   ---------------------------------SPAEKKEAYLCDITYSTNSEIGFDYLRDNMVVRAENMVQRPLNYALVDEVDSILIDEARTPLIVSGQTASDTSQLYHMADAYVKTLT   246
SECA1_STR_gordonii        160   ---------------------------------SPAEKREAYACDITYSTNSEIGFDYLRDNMVVRAENMVQRPLNYALVDEVDSILIDEARTPLIVSGPVSSETNQLYHMADSFVKSLN   246
SECA1_STR_pneumoniae      160   ---------------------------------SPMEKKEAYECDITYSTNSEIGFDYLRDNMVVRAENMVQRPLNYALVDEVDSILIDEARTPLIVSGANAVETSQLYHMADHYVKSLN   246
SECA1_STR_agalactiae      160   ---------------------------------SPFEKREAYNCDITYSTNAEVGFDYLRDNMVVRQEDMVQRPLNYALVDEVDSVLIDEARTPLIVSGPVSSEMNQLYTRADMFVKTLN   246
SECA1_LIS_monocytogenes   160   ---------------------------------SSTEKREAYACDITYSTNNELGFDYLRDNMVVYKEEMVQRPLAFAVIDEVDSILVDEARTPLIISGE-AEKSTILYVRANTFVRTLT   245
SECA1_LIS_innocua         160   ---------------------------------SSTEKREAYACDITYSTNNELGFDYLRDNMVVYKEEMVQRPLAFAVIDEVDSILVDEARTPLIISGE-AEKSTILYVRANTFVRTLT   245
SECA1_LIS_welshimeri_     160   ---------------------------------SSTEKREAYACDITYSTNNELGFDYLRDNMVVYKEEMVQRPLSFAVIDEVDSILVDEARTPLIISGE-AEKSTILYVRANTFVRTLT   245
SECA_BAC_subtilis         160   ---------------------------------SKDEKREAYAADITYSTNNELGFDYLRDNMVLYKEQMVQRPLHFAVIDEVDSILIDEARTPLIISGQ-AAKSTKLYVQANAFVRTLK   245
SECA1_BAC_anthracis       160   ---------------------------------SREEKQEAYAADITYSTNNELGFDYLRDNMVLYKEQCVQRPLHFAIIDEVDSILVDEARTPLIISGQ-AQKSTELYMFANAFVRTLE   245
SECA1_BAC_thuringiensis   160   ---------------------------------SREEKQEAYAADITYSTNNELGFDYLRDNMVLYKEQCVQRPLHFAIIDEVDSILVDEARTPLIISGQ-AQKSTELYMFANAFVRTLE   245
SECA1_BAC_cereus          160   ---------------------------------SREEKQEAYAADITYSTNNELGFDYLRDNMVLYKEQCVQRPLHFAIIDEVDSILVDEARTPLIISGQ-AQKSTELYMFANAFVRTLE   245
SECA1_STA_epidermidis_    166   ---------------------------------STEEKREAYAQDITYSTNNELGFDYLRDNMVNYAEERVMRPLHFAIIDEVDSILIDEARTPLIISGE-AEKSTSLYTQANVFAKMLK   251
SECA1_STA_haemolyticus    166   ---------------------------------STNEKREAYAQDITYSTNNELGFDYLRDNMVNYAEERVMRPLHFAIIDEVDSILIDEARTPLIISGE-AEKSTSLYTQANVFAKMLK   251
SECA1_STA_aureus          166   ---------------------------------TTEEKREAYAQDITYSTNNELGFDYLRDNMVNYSEDRVMRPLHFAIIDEVDSILIDEARTPLIISGE-AEKSTSLYTQANVFAKMLK   251
SECA1_ALK_metalliredigens 161   ---------------------------------TIEQRRAAYNADVTYGTNNEFGFDYLRDNMVIYQKDMVQREQNYAIVDEVDSILIDEARTPLIISGQ-GEKSTKLYHIVDQFVKTLK   246
SECA1_PEP_difficile       160   ---------------------------------NPKVRKEQYDCDITYGTNNEYGFDYLKDNMVIHKEQRVQRGLNYAIVDEVDSILIDEARTPLIISGP-GDKSTHLYSDANTFVLTLK   245
SECA2_PEP_difficile       160   ---------------------------------DPNIRKQQYKCDITYGTNSEFGFDYLRDNMVPDLSHKVQRELNFAIVDEVDSILIDEARTPLIIAGD-GDEDLKLYELANSFVKTVK   245
SECA2_model_opened_conf   148   ---------------------------------DPNIRKQQYKCDITYGTNSEFGFDYLRDNMVPDLSHKVQRELNFAIVDEVDSILIDEARTPLIIAGD-GDEDLKLYELANSFVKTVK   233
SECA_E_coli_              162   ---------------------------------PAPAKREAYAADITYGTNNEYGFDYLRDNMAFSPEERVQRKLHYALVDEVDSILIDEARTPLIISGP-AEDSSEMYKRVNKIIPHLI   247
SECA1_MYC_vanbaalenii     159   ---------------------------------TPDQRRAAYNADITYGTNWELGFDYLRDNMALRLEDCVQRGHHFAIVDEVDSILIDEARTPLIISGP-ADGGSNWYTEFARLAPLMK   244
SECA1_MYC_ulcerans        158   ---------------------------------TPEERRVAYNADITYGTNNEFGFDYLRDNMAHTLDDCVQRGHNFVIVDEVDSILIDEARTPLIISGP-ADGSSNWYTEFARLAPLME   243
SECA1_MYC_paratuberculosi 158   ---------------------------------TPDERRVAYNADITYGTNNEFGFDYLRDNMAHSLDDLVQRGHNFAIVDEVDSILIDEARTPLIISGP-ADGASNWYLEFARLAPLME   243
SECA1_MYC_tuberculosis    161   ---------------------------------TPDERRVAYNADITYGTNNEFGFDYLRDNMAHSLDDLVQRGHHYAIVDEVDSILIDEARTPLIISGP-ADGASNWYTEFARLAPLME   246
SECA1_MYC_bovis           161   ---------------------------------TPDERRVAYNADITYGTNNEFGFDYLRDNMAHSLDDLVQRGHHYAIVDEVDSILIDEARTPLIISGP-ADGASNWYTEFARLAPLME   246
SECA1_MYC_leprae          161   ---------------------------------TPDERRVAYNADITYGTNNEFGFDYLRDNMAHSLDDLVQRGHSYAIVDEVDSILIDEARTPLIISGS-ADGASNWYTEFARLVRLMD   246
SECA1_MYC_smegmatis       158   ---------------------------------TPDERRAAYAADITYGTNNEFGFDYLRDNMAHSVDDMVQRGHNFAIVDEVDSILIDEARTPLIISGP-ADGASHWYQEFARIVPMME   243
SECA1_COR_diphtheriae     160   ---------------------------------QPAERREAYNADITYGTNNELGFDYLRDNMVRSLDELVQRGHHYAIVDEVDSILIDEARTPLIISGP-VDGSSQWYSVFAQIVPRMT   245
SECA1_COR_efficiens       160   ---------------------------------RPAERKVAYDCDITYGTNNELGFDYLRDNMARSLNDLVQRGHHYAIVDEVDSILIDEARTPLIISGP-VDGSSQWYNVFAQIVPQLT   245
SECA1_COR_glutamicum      160   ---------------------------------RPDERREAYAADITYGTNNELGFDYLRDNMARSLSDLVQRGHNYAIVDEVDSILIDEARTPLIISGP-VDGTSQFYNVFAQIVPRMT   245
SECA1_COR_jeikeium        161   ---------------------------------NPAERREAYNADITYGTNNEFGFDYLRDNMAHSLNDLVQRGHNYAIVDEVDSILIDEARTPLIISGP-VEGSSKWFSAFAAIAPKLT   246
SECA1_THE_fusca           161   ---------------------------------SPAARRKAYQADITYGTNNEFGFDYLRDNMARSLDNCVQRGHHYAIVDEVDSILIDEARTPLIISGP-AEQNSRWYVEFAKIAPRLR   246
SECA2_MYC_vanbaalenii     158   ---------------------------------KPDERRAAYAADITYGTNNEFGFDYLRDNMAGRLEDRVQRGHCYAIVDEVDSILIDEARTPMIISGP-ADDATRWYAEFARLAALMT   243
SECA_THE_maritima_        172   EENWSVWPDGFNGEVLKEESMNKEAVEAFQVELKEITRKEAYLCDVTYGTNNEFGFDYLRDNLVLDYNDKVQRGHFYAIVDEADSVLIDEARTPLIISGP-SKESPSVYRRFAQIAKKFV   290
SECA2_GEO_kaustophilus    160   ---------------------------------TASEKKEAYAADITYGTGTEFGFDYLRDNMVYRLEDKVQRPLYYAIIDEIDSILIDEARTPLIIANK-SGIGAELFPIMARIVRTFE   245
SECA2_GEO_thermodenitrifi 161   ---------------------------------SPQEKKQAYQADITYGIGTEFGFDYLRDHMVYDASDKVQRPYHYAIIDEIDSVLIDEAKTPLIIAGK-TRSSTELHYIAARLVKRFE   246
SECA2_BAC_anthracis       161   ---------------------------------DPSEKKLAYEADITYGIGTEFGFDYLRDNMAASKNEQVQRPYHFAIIDEIDSVLIDEAKTPLIIAGK-KSSSSDLHYLCAKVIKSFQ   246
SECA2_BAC_thuringiensis   161   ---------------------------------DPFEKKLAYEADITYGIGTEFGFDYLRDNMAASKNEQVQRPYHFAIIDEIDSVLIDEAKTPLIIAGK-KSSSSDLHYLCAKVIKSFQ   246
SECA2_BAC_cereus          161   ---------------------------------DPSEKKLAYEADITYGIGTEFGFDYLRDNMAASKNEQVQRPYHFAIIDEIDSVLIDEAKTPLIIAGK-KSSSSDLHYLCAKVIKSFQ   246
SECA2_LIS_welshimeri      155   ---------------------------------DKDQKKAIYTADVIYGTASEFGFDYLRDNMVRQKEDKVQSGLDFVLIDEADSILIDEARTPLLISDR-KEEDLSLYQTANKLVKTML   240
SECA2_LIS_monocytogenes   155   ---------------------------------DIAQKKAIYTADVIYGTASEFGFDYLRDNMVRQKEDKVQSGLDFVLIDEADSILIDEARTPLLISDR-KEEDLSLYHTANKLVKKMM   240
SECA2_LIS_innocua         155   ---------------------------------DKDQKKAIYTADVIYGTASEFGFDYLRDNMVRQKEDKVQSGLDFVLIDEADSILIDEARTPLLISDR-KEEDLSLYQTANELVQTMM   240
SECA2_ALK_metalliredigens 165   ---------------------------------NLEERRKAYLKDITYVTARESGFDYLRDFLCTEVKDLVHRPFNYAILDEADSILIDEARIPLVIAGN-VSEEGFNYDHLIEIVRSLK   250
SECA2_LAC_johnsonii       155   -----------------------------KKKITAKTKRKWYNSDIVYTTASSLAFDYLFNNLASSKENQYLRPFNYVIVDEVDEVLLDEAQTPFVVSSS-PNVQSNLYHLADQFVRLLD   244
SECA2_STA_haemolyticus    161   ------------------------------YEYAENEKQMLYNHDIIYTTNGRLGFDYLFDNLADHINAKYLPELNFAIIDEVDSIILDAAQTPLVISGA-PRVQSNLFHIIKMFVETLV   249
SECA2_STA_aureus          161   ------------------------------YEYQKGEKRNIYEHDIIYTTNGRLGFDYLIDNLADSAEGKFLPQLNYGIIDEVDSIILDAAQTPLVISGA-PRLQSNLFHIVKEFVDTLI   249
SECA2_STA_epidermidis     161   ------------------------------YEYAENEKYELYHHDIVYTTNGRLGFDYLIDNLADDIRAKFLPKLNFAIIDEVDSIILDAAQTPLVISGA-PRVQSNLFHIVKTFVETLE   249
SECA2_STR_Agal            161   ------------------------------EDRDAATKRAVYSSDIVYSTSSALGFDYLIDNLASSKSQKYMPKLHYAIVDEADAVLLDMAQTPLVISGS-PRVQSNLYKIADELILSFE   249
SECA2_STR_Gordon          154   ------------------------------KPLETKEKRRIYQADIIYTTNSALGFDYLIENLAENKKNQFLREFNYVIIDEIDSILLDSAQTPLIISGA-PRVQSNFYDMMNTLVQTLY   242
SECA2_STR_Pneum           157   ------------------------------KEMKAEEKKLIYASDIIYTTNSNLGFDYLNDNLASNEEGKFLRPFNYVIIDEIDDILLDSAQTPLIIAGS-PRVQSNYYAIIDTLVTTLV   245
SECA2_STR_Sanguin         154   ------------------------------ENLTPKQKRLIYQADIVYTTNSALGFDYLIENLAENKDSQYLSPFNYVIIDEIDSILLDSAQVPLVISGA-PRVQSNFYSIMDTFITTLK   242
SECA2_COR_efficiens       158   ---------------------------------TPEQRREAYRCDVVYGSVSEIGFDVLRDQLITRRADAVQRRADVAIIDEADSVLVDEALVPLVLAGN-QPGHAPRGKITEVVRR-LK   242
SECA2_MYC_ulcerans        197   ---------------------------------TGADRRAAYECDVTYASVNEIGFDVLRDQLVTDVADLVSPNPDVALIDEADSVLVDEALVPLVLAGT-THRETPRLEIIKLVAQ-LV   281
SECA2_MYC_paratuberculosi 169   ---------------------------------SSEERRAAYGCDVTYASVNEIGFDVLRDQLVTDVADLVSPNPDVALIDEADSVLVDEALVPLVLAGT-THRETPRLEIIKLVGE-LE   253
SECA2_MYC_tuberculosis    199   ---------------------------------TPDERRTAYDRDVTYASVNEIGFDVLRDQLVTDVNDLVSPNPDVALIDEADSVLVDEALVPLVLAGT-THRETPRLEIIRLVAE-LV   283
SECA2_MYC_bovis           199   ---------------------------------TPDERRTAYDRDVTYASVNEIGFDVLRDQLVTDVNDLVSPNPDVALIDEADSVLVDEALVPLVLAGT-THRETPRLEIIRLVAE-LV   283
SECA2_MYC_leprae          169   ---------------------------------TREDRKAAYGCDVTYASVNEIGFDVLRDQLVTDVDDLVSPHPDVALIDEADSVLVDEALVPLVLAGA-THRETPRLEIIKLVGE-LS   253
SECA2_MYC_smegmatis       169   ---------------------------------TADERREAYQCDVTYASVNEIGFDVLRDQLVTDVADLVSPNPDVALIDEADSVLVDEALVPLVLAGT-SHREQPRVEIIRMVGE-LE   253
SECA2_THE_fusca           159   ---------------------------------TTEERRAAYAADITYASVSELGFDVLRDRLATDVSELVVPEPNVAIIDEADSVLVDEARVPLVLAGA-AEPAESDAAMAELVRR-LR   243
SECA2_COR_diphtheriae     158   ---------------------------------NTEQRRRAYASSIVYAPVTEIGFDVLRDQLVTQRSHAVQNGADVAIVDEADSVLIDEALVPLVLAGN-EPGTAPAGRITEIVRR-LK   242
SECA2_COR_glutamicum      158   ---------------------------------DAGERRQAYKAAIVYGPVNEIGFDVLRDQLITRREDAVQHGADVAIIDEADSVLVDEALVPLVLAGN-QPGHAPRGKITDVVRS-LK   242
SECA2_COR_jeikeium        166   ---------------------------------TADQRRDIYSRDVIFGAINELGFDVLRDQLITRRADQVRTPADVAVIDEADSVMVDEALVPLVLAGS-EPGPAPAGRITDLVKR-ME   250
Consensus_aa:                   .................................s..p++.hY..DlhYsosschGFDYLpDphh.p.pp.hb..hp@AllDEhDSlLlDEAphPLlltG..sp.ps..@..h..hhpph.
Consensus_ss:                                                     hhhhhhhhh  eeeeehhhhhhhhhhhhhh  hhhh      eeeee   eee       eee        hhhhhhhhhhhhhh
```

Highy conserved PPDX loop

```
Conservation:                                   65            5 5   9    5  5          575              5558   5  5 69 6       56 768  599 5 55    8 7696
SECA1_GEO_thermodenitrifi   246  KDV------------DYT----YDEKSKSVQLTEEGMNKAERAF-------GIDNLFDLKHV-TLNHHIQLALRAHVTMQRDVDYVVQ----DGKVIIVDPFTGRLMHGRRYSDGLHQAI  337
SECA1_GEO_kaustophilus      246  KDV------------DYT----YDEKTKSVQLTEEGINKAERAF-------GIDNLFDLKHV-TLNHHIQLALRAHVTMQRDVDYVVQ----DGKVIIVDPFTGRLMHGRRYSDGLHQAI  337
SECA1_LAC_johnsonii         246  EDKSDDDVDDDEDHGDYK----IDWPTKTINLTNQGIKKACEHF-------GLKNLYDIDNQ-VLVHHIDQALRANYIMLKDIDYVVQ----NGEVMIVDSFTGRVMEGRRYSDGLHQAI  349
SECA1_STR_parasanguinis     247  ED-------------DYI----IDVPSKTIGLSDSGIDKAESYF-------NLENLYDIENV-ALTHFIDNALRANYIMILDIDYVVSE---DQEILIVDQFTGRTMEGRRYSDGLHQAI  338
SECA1_STR_gordonii          247  KD-------------DYI----IDVPSKTIGLSDSGIDKAESYF-------KLDNLYDIENV-ALTHFIDNALRANYIMILDIDYVVSE---EQEILIVDQFTGRTMEGRRYSDGLHQAI  338
SECA1_STR_pneumoniae        247  KD-------------DYI----IDVQSKTIGLSDSGIDRAESYF-------KLENLYDIENV-ALTHFIDNALRANYIMLLDIDYVVSE---EQEILIVDQFTGRTMEGRRYSDGLHQAI  338
SECA1_STR_agalactiae        247  SD-------------DYI----IDVPTKTIGLSDTGIDKAENYF-------HLNNLYDLENV-ALTHYIDNALRANYIMLLNIDYVVSE---EQEILIVDQFTGRTMEGRRFSDGLHQAI  338
SECA1_LIS_monocytogenes     246  EEE------------DYT----VDIKTKSVQLTEDGMTKGENYF-------DVENLFDLENT-VILHHIAQALKANYTMSLDVDYVVQ----DDEVLIVDQFTGRIMKGRRFSEGLHQAL  337
SECA1_LIS_innocua           246  EEE------------DYT----VDIKTKSVQLTEDGMTKGENYF-------DVENLFDLENT-VILHHIAQALKANYTMSLDVDYVVQ----DDEVLIVDQFTGRIMKGRRFSEGLHQAL  337
SECA1_LIS_welshimeri_       246  EEK------------DYT----VDIKTKSVQLTEEGMTKGENYF-------DVENLFDLENT-VILHHIAQALKANYTMSLDVDYVAQ----DDEVLIVDQFTGRIMKGRRFSEGLHQAL  337
SECA_BAC_subtilis           246  AEK------------DYT----YDIKTKAVQLTEEGMTKAEKAF-------GIDNLFDVKHV-ALNHHINQALKAHVAMQKDVDYVVE----DGQVVIVDSFTGRLMKGRRYSEGLHQAI  337
SECA1_BAC_anthracis         246  NEK------------DYS----FDVKTKNVMLTEDGITKAEKAF-------HIENLFDLKHV-ALLHHINQALRAHVVMHRDTDYVVQ----EGEIVIVDQFTGRLMKGRRYSEGLHQAI  337
SECA1_BAC_thuringiensis     246  NEK------------DYS----FDVKTKNVMLTEDGITKAEKAF-------HIENLFDLKHV-ALLHHINQALRAHVVMHRDTDYVVQ----EGEIVIVDQFTGRLMKGRRYSEGLHQAI  337
SECA1_BAC_cereus            246  NEK------------DYS----FDVKTKNVMLTEDGITKAEKAF-------HIENLFDLKHV-ALLHHINQALRAHVVMHRDTDYVVQ----EGEIVIVDQFTGRLMKGRRYSEGLHQAI  337
SECA1_STA_epidermidis_      252  AED------------DYN----YDEKTKAVHLTEQGADKAERMF-------KVDNLYDVQNV-EVISHINTALRAHVTLQRDVDYMVV----DGEVLIVDQFTGRTMPGRRFSEGLHQAI  343
SECA1_STA_haemolyticus      252  AED------------DYK----YDEKTKAVHLTEQGADKAERMF-------KIENLYDVQNV-EVISHINTALKAHVTLQRDVDYMVV----DGEVLIVDQFTGRTMPGRRFSEGLHQAI  343
SECA1_STA_aureus            252  QDE------------DYK----YDEKTKAVHLTEQGADKAERMF-------KVENLYDVQNV-DVISHINTALRAHVTLQRDVDYMVV----DGEVLIVDQFTGRTMPGRRFSEGLHQAI  343
SECA1_ALK_metalliredigens   247  IED------------DVS----LDEKANSVTLTEDGGTKAEKAF-------GIENLADMNNM-ELSHHINQALKARNLMRLDKDYVVK----DGEIIIVDDFTGRLMFGRRYSDGLHQAI  338
SECA1_PEP_difficile         246  PD-------------DYE----LEEKDKAVSLTASGIQKAEVYF-------NVDNITDISHT-ELYHHINQALRAHVIMKKDVDYVAK----DGEIVIVDEFTGRLMFGRRYSEGLHQAI  336
SECA2_PEP_difficile         246  EE-------------DFE----LDRKDKTIALTASGISKAESFF-------GITNLTDIKNI-ELYHHINQALRGHKLMEKDVDYVIS----NGEVMIVDEFTGRVMDGRRYTDGLHQAI  336
SECA2_model_opened_conf     234  EE-------------DFE----LDRKDKTIALTASGISKAESFF-------GITNLTDIKNI-ELYHHINQALRGHKLMEKDVDYVIS----NGEVMIVDEFTGRVMDGRRYTDGLHQAI  324
SECA_E_coli_                248  RQEKEDSE-TFQGEGHFS----VDEKSRQVNLTERGLVLIEELLVKEGIMDEGESLYSPANI-MLMHHVTAALRAHALFTRDVDYIVK----DGEVIIVDEHTGRTMQGRRWSDGLHQAV  357
SECA1_MYC_vanbaalenii       245  PDV------------HYE----VDIKKRVVGINEAGVEFVEDQL-------GIENLYEAANS-PLISYLNNAIKAKELFERDKHYIVR----NGEVFIVDEFTGRMLVGRRYNEGLHQAI  336
SECA1_MYC_ulcerans          244  KDT------------HYE----VDLRKRTVGVHELGVEFVEDQL-------GIDNLYEAANS-PLVSYLNNALKAKELFNRDKDYIVR----NGEVLIVDEFTGRVLIGRRYSEGMHQAI  335
SECA1_MYC_paratuberculosi   244  KDV------------HYE----VDLRKRTVGVHELGVEFVEDQL-------GIDNLYEAANS-PLVSYLNNALKAKELFHRDKDYIVR----DGEVLIVDEFTGRVLYGRRYNEGMHQAI  335
SECA1_MYC_tuberculosis      247  KDV------------HYE----VDLRKRTVGVHEKGVEFVEDQL-------GIDNLYEAANS-PLVSYLNNALKAKELFSRDKDYIVR----DGEVLIVDEFTGRVLIGRRYNEGMHQAI  338
SECA1_MYC_bovis             247  KDV------------HYE----VDLRKRTVGVHEKGVEFVEDQL-------GIDNLYEAANS-PLVSYLNNALKAKELFSRDKDYIVR----DGEVLIVDEFTGRVLIGRRYNEGMHQAI  338
SECA1_MYC_leprae            247  KDV------------HYE----VDLRKRTVGVHEKGMEFVEDQL-------GIDNLYEVANS-PLVSYLNNALKAKELFNRDKDYIVR----DGEVLIVDEFTGRVLIGRRYNEGMHQAI  338
SECA1_MYC_smegmatis         244  KDV------------HYE----VDLRKRTVGVHELGVEFVEDQL-------GIDNLYEAANS-PLVSYLNNALKAKELFQRDKDYIVR----NGEVLIVDEFTGRVLMGRRYNEGMHQAI  335
SECA1_COR_diphtheriae       246  RDI------------HYE----VDNRKRTVGVREEGVAFVEDQL-------GIDNLYAPEHS-QLVSYLNNAIKAKELFNRDKDYIVR----NGEVLIVDDFTGRVLDGRRYNEGMHQAI  337
SECA1_COR_efficiens         246  RDI------------HYE----VDHRKKTVGIKEEGVEKVEDLL-------GIENLYAPEHS-QLVSYLNNAIKAEELFERDKDYIVR----NGEVMIVDGFTGRVLAGRRYNEGMHQAI  337
SECA1_COR_glutamicum        246  KDV------------HYE----VDERKKTVGVKEEGVEYVEDQL-------GIDNLYAPEHS-QLVSYLNNAIKAQELFTRDKDYIVR----NGEVMIVDGFTGRVLAGRRYNEGMHQAI  337
SECA1_COR_jeikeium          247  RDI------------HYE----VDERKKTVGVKEEGVEFVEDQL-------GIENLYAPEHS-QLVSYLNNSIKAKELFTRDKDYIVR----NGEVVIVDEFTGRILDGRRYNEGIHQAI  338
SECA1_THE_fusca             247  RDV------------DYE----VDEKKRTVGITEAGVAKVEDWL-------GIDNLYESVNT-PLISFLHNAIKAKELYRRDRDYIVK----DGEVLIVDEFTGRILRGRRYNEGMHQAI  338
SECA2_MYC_vanbaalenii       244  RDV------------HYE----VDTRRRTIGVSEAGVALVEDQL-------GVDNLYQVVHA-PLVGHLNNAVRAKELFHRDREYIVND---DGEVLIVDEFTGRVLVGRRYNEGLHQAI  336
SECA_THE_maritima_          291  KDK------------DFT----VDEKARTIILTEEGVAKAEKII-------GVENLYDPGNV-SLLYHLINALKALHLFKKDVDYVVM----NGEVIIVDEFTGRLLPGRRYSGGLHQAI  382
SECA2_GEO_kaustophilus      246  EGK------------EYE----RSLETKQIFLTEEGAQKIERAF-------GIDNLYDWEHH-VLLHHAMQSLRAWFIMRRDVDYIVK----NGKVMIVDPFTGRVMEGRSFSDGLHQAI  337
SECA2_GEO_thermodenitrifi   247  REV------------DYI----YDGETKTVNLTDEGIEKVEKAF-------GIDNLYDAEHQ-VLYHYVIQALRAHVLFQRDVDYIIR----DGKVLLVDAFTGRVMEGRSLSDGLHQAI  338
SECA2_BAC_anthracis         247  DTL------------HYT----YDAESKSASFTEDGITKIEDLF-------DIDNLYDLEHQ-TLYHYMIQALRAHVAFQCDVDYIVH----DEKILLVDIFTGRVMDGRSLSDGLHQAL  338
SECA2_BAC_thuringiensis     247  DTL------------HYT----YDAESKSASFTEDGIIKIEDLF-------DIDNLYDLEHQ-TLYHYMIQALRAHVAFQCDVDYIVH----DEKILLVDIFTGRVMDGRSLSDGLHQAL  338
SECA2_BAC_cereus            247  DTL------------HYT----YDAESKSASFTEDGITKIEDLF-------DIDNLYDLEHQ-TLYHYMIQALRAHVAFQCDVDYIVH----DEKILLVDIFTGRVMDGRSLSDGLHQAL  338
SECA2_LIS_welshimeri        241  KD-------------EYE----IEEHKRFVWLNDAGIEKAQKFW-------GVESLYSAEGQ-AELRITMLLMRAHFLMHKDKDYVVL----DDEVLIIDPHTGRALPGRRFNDGLHQAI  331
SECA2_LIS_monocytogenes     241  KD-------------DYE----MEEHKRFVWLNDAGIEKAQKFW-------GVESLYSAEAQ-SELRITMLLMRAHFLMHKDKDYVVL----DDEVLIIDPHTGRALPGRRFNDGLHQAI  331
SECA2_LIS_innocua           241  KD-------------DYE----IEEHKRFVWLNDAGIERAQKFW-------GVESLYSAEAQ-VELRITMLLMRAHFLMHKDKDYVVL----DGEVLIIDPHTGRALPGRRFNDGLHQAI  331
SECA2_ALK_metalliredigens   251  PNV------------DYE----IDQHHHNVVLTEKGLIKIEKVS-------GCGNLYASENL-QLLTRLNCVLHAEILLKKEIDYIVR----KGKIEIIDEFTGRVADKRHWPDSLHEAV  342
SECA2_LAC_johnsonii         245  PEV------------DYV----FKKDDQLFWLTAHGIEKAEQFF-------KLDSLFSNESR-SVYRHIILAMGAHLTMRRGHDYLVV----KGEVVLLDEDSGRLKRGVQVSTGIHQAV  336
SECA2_STA_haemolyticus      250  EDE------------HFK----LNVNKKEVWLTDEGIDVANHYF-------KVNNIYLPQYF-DLVRVINLSLRAKYLFKDNLDYFIY----NGEVVLIDRITGRMLPGTKLQSGLHQAI  341
SECA2_STA_aureus            250  EDV------------HFK----MKKTKKEIWLLNQGIEAAQSYF-------NVEDLYSEQAM-VLVRNINLALRAQYLFESNVDYFVY----NGDIVLIDRITGRMLPGTKLQAGLHQAI  341
SECA2_STA_epidermidis       250  KDK------------DFI----VNFNKKEVWLTDEGSEKASHYF-------KVNSIYQQQYF-DLVRMIHLSLRAKYLFKYNLDYFIF----DGEIVLIDRITGRMLPGTKLQSGLHQAI  341
SECA2_STR_Agal              250  EQV------------DYY----FDKEHQEVWIKNQGVREAERYF-------RIPHFYKQSNR-ELVRHLNLSLKAHKLFERGKDYVVD----DGEIKLLDATNGRVLEGTKLQGGVHQAI  341
SECA2_STR_Gordon            243  EDE------------DYC----YDDEKNEVWLTQKGIRAAESFL-------GLNHLFSKENQ-ELVRHLNLALRAHMVYKKDQDYVVRQSENEAEVVLLDRATGRLMELTRLQGGQHQAI  338
SECA2_STR_Pneum             246  EGE------------DYI----FKEEKEEVWLTTKGAKSAENFL-------GIDNLYKEEHA-SFARHLVYAIRAHKLFTKDKDYIIR----GNEMVLVDKGTGRLMEMTKLQGGLHQAI  337
SECA2_STR_Sanguin           243  EEE------------DYH----YDDEKNEVWLTSKGILAAESFL-------DLEHLFSKENQ-ELVRHLNLALRAHKLYKKDKDYVVRQGDKEAEVVLLDRATGRLLEMTRLQGGQHQAI  338
SECA2_COR_efficiens         243  END------------HYT----VSEDRRNVFLTDKGAAALEQAL-------GITSLYDDEHVGTTLVQVNLALHAQALLIRDIHYIVR----DGKVQLIDASRGRVADLQRWPDGLQAAV  335
SECA2_MYC_ulcerans          282  KDK------------DADEYFATDADSRNVHLTEAGARKVEKAL-------GGIDLYSEEHVGTTLTEVNVALHAHVLLQRDVHYIVR----DDAVHLINASRGRIAQLQRWPDGLQAAV  378
SECA2_MYC_paratuberculosi   254  AGT------------DYD----TDADSRNVHLTDVGARKVEKAL-------GGIDLYSEEHVGTTLTEVNVALHAHVLLQRDVHYIVR----DDAVHLINASRGRIAQLQRWPDGLQAAV  346
SECA2_MYC_tuberculosis      284  GDK------------DADEYFATDSDNRNVHLTEHGARKVEKAL-------GGIDLYSEEHVGTTLTEVNVALHAHVLLQRDVHYIVR----DDAVHLINASRGRIAQLQRWPDGLQAAV  380
SECA2_MYC_bovis             284  GDK------------DADEYFATDSDNRNVHLTEHGARKVEKAL-------GGIDLYSEEHVGTTLTEVNVALHAHVLLQRDVHYIVR----DDAVHLINASRGRIAQLQRWPDGLQAAV  380
SECA2_MYC_leprae            254  AES------------DYD----TDSDSRNVHLTDVGARKVEKAL-------GGIDLYSEEHVGTTLTEVNVALHAHVLLQRDVHYIVR----DDAVHLVNASRGRIAQLQRWPDGLQAAV  346
SECA2_MYC_smegmatis         254  AGK------------HYD----TDAESRNVHLTEAGARVMEAKL-------GGIDLYSEEHVGTTLTEINVALHAHVLLQRDVHYIVR----DDAVHLINASRGRIASLQRWPDGLQAAV  346
SECA2_THE_fusca             244  PGI------------DYK----VDDDGRNVHLTDTGINVVEKAL-------GGVDLFSAEDT-TLLSRVNLALHAHALLHRDVHYVVR----DGKVRLINESRGRIALLQRWPDGLQAAV  335
SECA2_COR_diphtheriae       243  ENE------------HYS----VDADRRNVSLNDKGAALLEQVL-------GIQSLYDDAHIGTTLVQVNLALHAQALLIRDVHYIIR----DGKIALIDASKGRVAQLQRWPDGVQAAV  335
SECA2_COR_glutamicum        243  END------------DYT----IDDDRRNVFLTDKGAAKLEQQL-------GISSLYDDEHVGSTLVQVNLALHAQALLIRDIHYIVR----DSKVLLIDASRGRVADLQRWPDGLQAAV  335
SECA2_COR_jeikeium          251  EDK------------HFH----VSEDHRNVFLTDEGAAFVEKEL-------GVDSLYEDEG--ELLVQVNLALHAEHLLIRDVHYIVR----DGKVALIDGSRGRVAELQRWPDGLQAAV  341
Consensus_aa:                    .p.............c@.....hc.cp+pl.lsppGh..hEphh........lpsL@p.pp...hhp.l..tl+Ap.lhpc-.cYlV.....ssclhllD..sGRhh..p+hspGlHpAl
Consensus_ss:                                  ee    e     eeee hhhhhhhhhh                 hhhh hhhhhhhhhhhhhhhhh   eeeee      eeeeee               hhhhhhh
```

```
Conservation:                   9889 5       5 6568 8555 57   6998887     77   9 5 8  7766    9 9  8 67     8  5    6      569 9657  6  77  6  8    6
SECA1_GEO_thermodenitrifi  338   EAKEGLEIQNESMTLATITFQNYFRMYEKLAGMTGTAKTEEEEFRNIYNMRVVVIPTNRPVIREDRPDLIYRTMEGKFRAVVEDIAQRHAKGQPVLVGTVAIETSELLSEMLKKRGIPHN   457
SECA1_GEO_kaustophilus     338   EAKEGLEIQNESMTLATITFQNYFRMYEKLAGMTGTAKTEEEEFRNIYNMRVVVIPTNRPVIREDRPDLIYRTMEGKFRAVVEDIAARHAKGQPVLVGTVAIETSEMLSEMLKKRGIPHN   457
SECA1_LAC_johnsonii        350   EAKEGVKIQEESKTQATITYQNFFRMYKKLAGMTGTAKTEEEEFREIYNMEVITIPTNRPIARKDLPDILYPTLDSKFEAVVKEIKERHAKGQPVLVGTVAIESSERLSQMLNQAGIPHA   469
SECA1_STR_parasanguinis    339   EAKEGVPVQEETKTSASITYQNLFRMYKKLSGMTGTGKTEEEEFREIYNIRVIPIPTNRPIARIDHPDLLYPSLKSKFKAVVEDVKSRHEKGQPVLVGTVAVETSDYLSQLLVQAGVPHE   458
SECA1_STR_gordonii         339   EAKEGVPVQDETKTSASITYQNLFRMYKKLSGMTGTAKTEEEEFRETYNIRVIPIPTNRPIARIDHEDLLYPSLESKFKAVVEDVKERHLKGQPVLVGTVAVETSDYLSKKLVAAGIPHE   458
SECA1_STR_pneumoniae       339   EAKEGVPIQDETKTSASITYQNLFRMYKKLSGMTGTGKTEEEEFREIYNIRVIPIPTNRPVQRIDHSDLLYASIESKFKAVVEDVKARYQKGQPVLVGTVAVETSDYISKKLVAAGVPHE   458
SECA1_STR_agalactiae       339   EAKESVPIQEESKTSASITYQNMFRMYHKLAGMTGTGKTEEEEFREIYNMRVIPIPTNRPVQRIDHSDLLYPTLDSKFRAVVADVKERYEQGQPVLVGTVAVETSDLISRKLVAAGVPHE   458
SECA1_LIS_monocytogenes    338   EAKEGVTIQNESKTMATITFQNYFRMYKKLAGMTGTAKTEEEEFRDIYNMRVIEIPTNKVIIRDDRPDLIYTTIEAKFNAVVEDIAERHAKGQPVLVGTVAIETSELISSKLKRKGIKHD   457
SECA1_LIS_innocua          338   EAKEGVTIQNESKTMATITFQNYFRMYKKLAGMTGTAKTEEEEFRDIYNMRVIEIPTNKVIIRDDRPDLIFTTIEAKFNAVVEDIADRHAKGQPVLVGTVAIETSELISSKLKRKGIKHD   457
SECA1_LIS_welshimeri_      338   EAKEGVTIQNESKTMATITFQNYFRMYKKLAGMTGTAKTEEEEFRDIYNMRVIEIPTNKVIIRDDRPDLIFTTINAKFDAVVEDIAERNAKGQPVLVGTVAIETSELISSKLKRKGIKHD   457
SECA_BAC_subtilis          338   EAKEGLEIQNESMTLATITFQNYFRMYEKLAGMTGTAKTEEEEFRNIYNMQVVTIPTNRPVVRDDRPDLIYRTMEGKFKAVAEDVAQRYMTGQPVLVGTVAVETSELISKLLKNKGIPHQ   457
SECA1_BAC_anthracis        338   EAKEGVEIQNESMTLATITFQNYFRMYEKLSGMTGTAKTEEEEFRNIYNMNVIVIPTNKPIIRDDRADLIFKSMKGKFNAVVEDIVNRHKQGQPVLVGTVAIETSELISKMLTRKGVRHN   457
SECA1_BAC_thuringiensis    338   EAKEGVEIQNESMTLATITFQNYFRMYEKLSGMTGTAKTEEEEFRNIYNMNVIVIPTNKPIIRDDRADLIFKSMEGKFNAVVEDIVNRHKQGQPVLVGTVAIETSELISKMLTRKGVRHN   457
SECA1_BAC_cereus           338   EAKEGVEIQNESMTLATITFQNYFRMYEKLSGMTGTAKTEEEEFRNIYNMNVIVIPTNKPIIRDDRADLIFKSMEGKFNAVVEDIVNRHKQGQPVLVGTVAIETSELISKMLTRKGVRHN   457
SECA1_STA_epidermidis_     344   EAKEGVAIQNESKTMASITFQNYFRMYNKLAGMTGTAKTEEEEFRNIYNMTVTQIPTNKPVQRKDNSDLIYISQKGKFDAVVEDVVEKHKKGQPVLLGTVAVETSEYISNLLKKRGVRHD   463
SECA1_STA_haemolyticus     344   EAKEGVKIQNESKTMASITFQNYFRMYNKLAGMTGTAKTEEEEFRNIYNMTVTQIPTNKPVQRDDKSDLIYISQKGKFDAVVEDVVEKHKQGQPVLLGTVAVETSEYISNLLKKRGIRHD   463
SECA1_STA_aureus           344   EAKEGVQIQNESKTMASITFQNYFRMYNKLAGMTGTAKTEEEEFRNIYNMTVTQIPTNKPVQRNDKSDLIYISQKGKFDAVVEDVVEKHKAGQPVLLGTVAVETSEYISNLLKKRGIRHD   463
SECA1_ALK_metalliredigens  339   EAKEGLQIQRESKTLATITFQNYFRMYRKLSGMTGTAKTEEDEFRAIYNMDVVEIPTNRVIVRDDQADGVYKGEQGKFEALAKDIEGRYKKGQPVLVGTISIEKSEELATLLKRKGIPCE   458
SECA1_PEP_difficile        337   EAKEGLKIQRESKTLATVTFQNYFRMYKKLSGMTGTAKTEEEEFKAIYKMDVFQVPTNKLMIREDLPDCVYKSEIGKFNAVAQEIIERHKVNQPILVGTVSIEKSELLSQILKKKGIKHE   456
SECA2_PEP_difficile        337   EAKEGVEIKNESKTMATVTYQNFFRLYEKLSGMTGTAKTEEGEFESIYKLNVVQIPTNRPVIRADLHDKVFKTEEEKYSAVVEEIIRIHKTRQPILVGTVSVEKSEKLSKMLKKQGIKHQ   456
SECA2_model_opened_conf    325   EAKEGVEIKNESKTMATVTYQNFFRLYEKLSGMTGTAKTEEGEFESIYKLNVVQIPTNRPVIRADLHDKVFKTEEEKYSAVVEEIIRIHKTRQPILVGTVSVEKSEKLSKMLKKQGIKHQ   444
SECA_E_coli_               358   EAKEGVQIQNENQTLASITFQNYFRLYEKLAGMTGTADTEAFEFSSIYKLDTVVVPTNRPMIRKDLPDLVYMTEAEKIQAIIEDIKERTAKGQPVLVGTISIEKSELVSNELTKAGIKHN   477
SECA1_MYC_vanbaalenii      337   EAKEHVEIKAENQTVAQVTLQNYFRMYEKLAGMTGTAETEAAELHEIYKLGVVPIPTNRPMVRKDQSDLIYKTEEAKYIAVVDDVAERYEKGQPVLIGTTSVERSEFLSRQFEKRRIPHN   456
SECA1_MYC_ulcerans         336   EAKEHVEIKAENQTLATITLQNYFRLYNKHAGMTGTAQTEAAELHEIYKLGVVPIPTNRPMVREDQSDLIYKTEEAKYIAVVDDVAERYEKGQPVLIGTTSVERSEYLSRQFTKRRIPHN   455
SECA1_MYC_paratuberculosi  336   EAKEHVEIKAENQTLATITLQNYFRLYDKLSGMTGTAQTEAAELHEIYKLGVVPIPTNKPMIRTDQSDLIYKTEEAKYIAVVDDVVERYQKGQPVLIGTTSVERSEYLSRQFQKRRIPHN   455
SECA1_MYC_tuberculosis     339   EAKEHVEIKAENQTLATITLQNYFRLYDKLAGMTGTAQTEAAELHEIYKLGVVSIPTNMPMIREDQSDLIYKTEEAKYIAVVDDVAERYAKGQPVLIGTTSVERSEYLSRQFTKRRIPHN   458
SECA1_MYC_bovis            339   EAKEHVEIKAENQTLATITLQNYFRLYDKLAGMTGTAQTEAAELHEIYKLGVVSIPTNMPMIREDQSDLIYKTEEAKYIAVVDDVAERYAKGQPVLIGTTSVERSEYLSRQFTKRRIPHN   458
SECA1_MYC_leprae           339   EAKEHVEIKAENQTLATITLQNYFRLYDKLAGMTGTAQTEAAELHEIYKLGVVAIPTNKPMIREDRSDLIYKTEEAKYMAVVDDVAERYEKGQPVLIGTTSVERSEYLSRQFTKRHIPHN   458
SECA1_MYC_smegmatis        336   EAKERVEIKAENQTLATITLQNYFRLYDKLSGMTGTAETEAAELHEIYKLGVVPIPTNKPMVRQDQSDLIYKTEEAKFLAVVDDVAERHAKGQPVLIGTTSVERSEYLSKMLTKRRVPHN   455
SECA1_COR_diphtheriae      338   EAKENVEIKNENQTLATITLQNYFRLYDKLSGMTGTAETEASELHQIYKLDVIPIPTNRPNQREDMTDLVYKTQEAKFAAVVDDISERVTKGQPVLVGTTSVERSEYLSQLLQRRGIKHS   457
SECA1_COR_efficiens        338   EAKEKVEIKNENQTLATVTLQNYFRLYEKLSGMTGTAETEAAELHQIYKLDVIQIPTNRENQRDDLTDLVYKTQEAKFAAVVDDIAERIANGQPVLVGTVSVERSEYLSQLLTRRGIKHN   457
SECA1_COR_glutamicum       338   EAKERVEIKNENQTLATVTLQNYFRLYTKLAGMTGTAETEAAELNQIYKLDVIAIPTNRPNQREDLTDLVYKTQEAKFAAVVDDIAERTEKGQPVLVGTVSVERSEYLSQLLTKRGIKHN   457
SECA1_COR_jeikeium         339   EAKEHVEIKNENQTLATVTLQNYFRLYDKLAGMTGTAETEAAELKSTYKLDVAAIPTNKENKRKDNVDLIYKTQEAKFEAVAEDIAERVEIGQPVLVGTTSVERSEYLSRLLQRRGIKHN   458
SECA1_THE_fusca            339   EAKEKVKIKEENQTLAKITLQNYFRLYEKLAGMTGTAVTEAAEFQQTYNLGVVPIPTNKPMIREDLRDLVYKTEEAKFQAIVEDIAECHERGQPVLVGTTSVEKSELLSKMLKRRGIPHE   458
SECA2_MYC_vanbaalenii      337   EAKEGVEVKPENQTLATITLQNYFRLYDRLAGMTGTARSEASEFRDIYRLGVITIPPNRPVVRRDEVDVVYKTESAKFDAVVEDVVGRHAAGQPVLIGTTSVEKSEYLSGRLTERRVPHT   456
SECA_THE_maritima_         383   EAKEGVPIKEESITYATITFQNYFRMYEKLAGMTGTAKTEESEFVQVYGMEVVVIPTHKPMIRKDHDDLVFRTQKEKYEKIVEEIEKRYKKGQPVLVGTTSIEKSELLSSMLKKKGIPHQ   502
SECA2_GEO_kaustophilus     338   EAKEGVEITEENDIQATITIQNYFRMYEKLAGMTGSATPSKEEFWQTYRLRVITIPTNQPSRRTDWDDLVYQTYEAKVRKIIDEVKKMNAIGRPVLIGTTSVAQSEQLSAAFSKAGIPHH   457
SECA2_GEO_thermodenitrifi  339   EAKEGLEITEENKTYASITIQNYFRMYPILSGMTGTAKTEEKEFQRIYGIDVIPIPTNRPKIRVDLPDRVYMTRHDKYVAVAKEVKRRHESGQPVLIGTTSILQSEEVAKYLDQEQVPYE   458
SECA2_BAC_anthracis        339   EAKEGLEITEENQTQASITIQNFFRMYPALSGMTGTAKTEEKEFNRVYNMEVMPIPTNRPIIREDKKDVVYVTADAKYKAVREDVLKHNKQGRPILIGTMSILQSETVARYLDEANITYQ   458
SECA2_BAC_thuringiensis    339   EAKEGLEITEENQTQASITIQNFFRMYPALSGMTGTAKTEEKEFNRVYNMEVIPIPTNRPIIREDKKDVVYVTADAKYKAVREDVLKHNKQGRPILIGTMSILQSETVARYLDEANITYQ   458
SECA2_BAC_cereus           339   EAKEGLEITEENQTQASITIQNFFRMYPALSGMTGTAKTEEKEFNRVYNMEVIPIPTNRPIIREDKKDVVYVTADAKYKAVREDVLKHNKQGRPILIGTMSILQSETVARYLDEANITYQ   458
SECA2_LIS_welshimeri       332   EAKEEVEVKEESRTLATITIQNYFRMYKKISGMTGTAKTEEEEFRQIYNMDVVVIPTNLRINREDVPDDIFYTKKEKGRAIVYEVSWRYEKGQPTLIGTSSIKSNEWISGLLDAAGIPHQ   451
SECA2_LIS_monocytogenes    332   EAKEGVEVKEESRTLATITIQNYFRMYKKISGMTGTAKTEEEEFRQIYNMDVVVIPTNLRVNREDMQDDIFYTKKEKGRAIVYEVSWRYEKGQPTLIGTSSIKSNEWISGLLDAAGIPHQ   451
SECA2_LIS_innocua          332   EAKEGVEVKEESRTLATITIQNYFRMYKRLSGMTGTAKTEEEEFRQIYNMDVVVIPTNLRVNREDMPDDIFYTKKEKGRAIVYEVSWRYEKGQPTLIGTSSIKSNEWISSLLDAAGIPHQ   451
SECA2_ALK_metalliredigens  343   EAKEGLVSETKGRIMGSIALQYFLKLYPKLAGMTGTAISVMNELDRMYQLKVVLIPTNKPCIRKDYPDKIFTCKEIKEKTLILEIKNINATGQPILIGTSSVEESERIATTLNTEGIKCN   462
SECA2_LAC_johnsonii        337   EAKEKVELTKIQKTAASITFPALFALFNKVSGMSGTAKVNEEEFLQTYNLKVVTIPTRVPVIRKDYRPLIFLTTIDKLMTAVDDVVEMHKTGRPVLLVAGSVENSEIISELLLNIGIPHN   456
SECA2_STA_haemolyticus     342   EAKEGVELSQDLSVMATITFQNLFKLFNGFSGMTGTGKLGEKEFFDLYSKLVVEIPTNHPIIRNDKEDRVYAKSDEKNKAILEKVKEIHATKQPVLLITRTAEAAEYFSTQLFKDNIPNN   461
SECA2_STA_aureus           342   EAKEGMEVSTDKSVMATITFQNLFKLFESFSGMTATGKLGESEFFDLYSKIVVQAPTDKAIQRIDEPDKVFRSVDEKNIAMIHDIVELHETGRPVLLITRTAEAAEYFSKVLFQMDIPNN   461
SECA2_STA_epidermidis      342   EALENVEISQDMSVMATITFQNLFKQFDEFSGMTGTGKLGEKEFFDLYSKVVIEIPTHSPIERDDRPDRVFANGDKKNDAILKTVIGIHETQQPVLLITRTAEAAEYFSAELFKRDIPNN   461
SECA2_STR_Agal             342   EQKEHLNVTPESRAMASITYQNLFRMFTKLAGMTGTGKTAEKEFIEVYDMEVVRIPTNSPVRRIDYPDKIYTTLPEKIYATIEFVKQVHDTGQPILLVAGSVRMSELFSELLLLSGIPHS   461
SECA2_STR_Gordon           339   EAKEHVKLTQETRAMASITYQNLFKLFRKLSGMTGTGKVVESEFLETYSMSVVKIPTNCPVIRRDYPDQIYQTLPEKVFASLDEVKHYHAKGNPLLIFTGSVEMSEIYSSLLLREGIAHN   458
SECA2_STR_Pneum            338   EAKEHVKLSPETRAMASITYQSLFKMFNKISGMTGTGKVAEKEFIETYNMSVVRIPTNRPRQRIDYPDNLYITLPEKVYASLEYIKQYHAKGNPLLVFVGSVEMSQLYSSLLFREGIAHN   457
SECA2_STR_Sanguin          339   EAKEHVKLTEETRAMASITYQNLFRLFRKISGMTGTGKVVESEFMETYSMSVIKIPTNQPVIRQDLPDQLYQTLPEKVFASLDEVKHYHAQGNPLLIFTGSVEMSEIYSSLLLREGIAHN   458
SECA2_COR_efficiens        336   EAKEGLAVTEGGRILDTITLQALVGRYPMVCGMTGTAVEATDQLRTFYNLHVSVIERNNPLRRFDEADRIYATMAEKNRAIIEEIAHLHHTGQPVLVGTHDVAESEELADALRDLDIEVS   455
SECA2_MYC_ulcerans         379   EAKEGIETTETGEVLDTITVQALINRYVTVCGMTGTALAAGEQLRQFYKLGVSPIPPNTPNIREDESDRVYITAAAKNDAIVEHIAEVHDTGQPVLVGTRDVAESEDLHERLLRRDIPAV   498
SECA2_MYC_paratuberculosi  347   EAKEGIETTETGEVLDTITVQALINRYATVCGMTGTALAAGEQLRQFYKLGVSPIPPNKPNIREDEADRVYITAAAKNDAIVEHIIEVHETGQPVLVGTRDVAESEELHERLLRRGVPAV   466
SECA2_MYC_tuberculosis     381   EAKEGIETTETGEVLDTITVQALINRYATVCGMTGTALAAGEQLRQFYQLGVSPIPPNKPNIREDEADRVYITTAAKNDGIVEHITEVHQRGQPVLVGTRDVAESEELHERLVRRGVPAV   500
SECA2_MYC_bovis            381   EAKEGIETTETGEVLDTITVQALINRYATVCGMTGTALAAGEQLRQFYQLGVSPIPPNKPNIREDEADRVYITTAAKNDGIVEHITEVHQRGQPVLVGTRDVAESEELHERLVRRGVPAV   500
SECA2_MYC_leprae           347   EAKEGIETTETGEVLDTITVQALINRYATVCGMTGTALAAGEQLRQFYKLGVSPIPPNTPNIRDDASDRVYITAAAKNDAIVAHLAEVHETGQPVLVGTRNVAESEELHERLLRHGVPAV   466
SECA2_MYC_smegmatis        347   EAKEGIETTETGEVLDTITVQALINRYPRVCGMTGTALAAGEQLRQFYKLGVSPIPPNTPNIRKDEPDRVYITAAAKIDAIVEHIAEVHKTGQPVLVGTHDVAESEELHEKLLKAGVPAV   466
SECA2_THE_fusca            336   EAKEHLTPSETGEVLDSITVQSLVLRYPIRCGMTGTAMAVAEQLREFYELEVAVIAPNKPNIRIDEEDRLYATAEEKEEAVVEKVKEVHATGRPILIGTQDVAESERLAKRLRRAGLECV   455
SECA2_COR_diphtheriae      336   EAKEGLVVTEGGRILDTLTLQSLMGRYPIVCGMTGTAVEATDQLRQFYDLRVSVIEPHKQSQRFDEADRVYATQAEKFRALVKEIELLHTTGQPVLIGTSDVSESEELAQALQARDITVN   455
SECA2_COR_glutamicum       336   EAKEGLAVSEGGKILDTITLQALIGRYPMACGMTGTAVEATDQLRTFYDLHVSVIERNHPLKRFDEADRIYATMAEKNRAIIDEIALLHSTGQPVLVGTHDVAESEELATALRELNIEVS   455
SECA2_COR_jeikeium         342   EAKEGLDVTDGGRILDQITIQALVGMYPEVCGMTGTALAAGDQLRQFYNLQVSVIEPNVPNIRFDEADRVYVSAAERNDAVVKHIVEVQKTGQPQLVGTQDVAESEELAEALLSAGVECS   461
Consensus_aa:                    EAKE.lplpppsphhtoIThQshhpb@.pltGMTGTtbh..pEh.phYph.Vh.IPsNpP..R.D..D.l@.o...K..Allccl.p.@.pGpPlLlsT.sl.pSEbltp.L.p.sl.hp
Consensus_ss:                    hhhh        eeeee hhhhhhhhhhhhh     hhhhhhhhhhh    eeee     eeeeee eeeee hhhhhhhhhhhhhhhh     eeeee  hhhhhhhhhhhhh    ee
```

```
Conservation:                      69895    99  75 79  5 67679 89999898 97                                                               7   999 768 575 5
SECA1_GEO_thermodenitrifi  458  VLNAKNHAKEAEIIAQAGQKGAVTIATNMAGRGTDIKLGE-------------------------------------------------------------GVKELGGLAVIGTERHESR  516
SECA1_GEO_kaustophilus     458  VLNAKNHAKEAEIIAQAGQKGAVTIATNMAGRGTDIKLGE-------------------------------------------------------------GVKELGGLAVIGTERHESR  516
SECA1_LAC_johnsonii        470  VLNAKNHAKEAEIIMNAGQRGAVTIATNMAGRGTDIKLGP-------------------------------------------------------------GVKELGGLAVIGTERHESR  528
SECA1_STR_parasanguinis    459  VLNAKNHYKEAQIIMNAGQRGAVTIATNMAGRGTDIKLGE-------------------------------------------------------------GVRELGGLCVIGTERHESR  517
SECA1_STR_gordonii         459  VLNAKNHYREAQIIMNAGQRGAVTIATNMAGRGTDIKLGE-------------------------------------------------------------GVRELGGLCVIGTERHESR  517
SECA1_STR_pneumoniae       459  VLNAKNHYREAQIIMNAGQRGAVTIATNMAGRGTDIKLGE-------------------------------------------------------------GVRELGGLCVIGTERHESR  517
SECA1_STR_agalactiae       459  VLNAKNHFKEAQIIMNAGQRGAVTIATNMAGRGTDIKLGE-------------------------------------------------------------GVRELGGLCVIGTERHESR  517
SECA1_LIS_monocytogenes    458  VLNAKQHEREADIIKHAGERGAVVIATNMAGRGTDIKLGE-------------------------------------------------------------GTIEAGGLAVIGTERHESR  516
SECA1_LIS_innocua          458  VLNAKQHEREADIIKNAGERGAVTIATNMAGRGTDIKLGE-------------------------------------------------------------GTIDAGGLAVIGTERHESR  516
SECA1_LIS_welshimeri_      458  VLNAKQHEREADIIKHAGERGAVTIATNMAGRGTDIKLGE-------------------------------------------------------------GTIEAGGLAVIGTERHESR  516
SECA_BAC_subtilis          458  VLNAKNHEREAQIIEEAGQKGAVTIATNMAGRGTDIKLGE-------------------------------------------------------------GVKELGGLAVVGTERHESR  516
SECA1_BAC_anthracis        458  ILNAKNHAREADIIAEAGMKGAVTIATNMAGRGTDIKLGD-------------------------------------------------------------DIKNI-GLAVIGTERHESR  515
SECA1_BAC_thuringiensis    458  ILNAKNHAREADIIAEAGMKGAVTIATNMAGRGTDIKLGD-------------------------------------------------------------DIKNI-GLAVIGTERHESR  515
SECA1_BAC_cereus           458  ILNAKNHAREADIIAEAGMKGAVTIATNMAGRGTDIKLGD-------------------------------------------------------------DIKNI-GLAVIGTERHESR  515
SECA1_STA_epidermidis_     464  VLNAKNHEREAEIVSNAGQKGAVTIATNMAGRGTDIKLGD-------------------------------------------------------------GVEELGGLAVIGTERHESR  522
SECA1_STA_haemolyticus     464  VLNAKNHEREAEIVANAGQKGAVTIATNMAGRGTDIKLGE-------------------------------------------------------------GVEEIGGLAVIGTERHESR  522
SECA1_STA_aureus           464  VLNAKNHEREAEIVAGAGQKGAVTIATNMAGRGTDIKLGE-------------------------------------------------------------GVEELGGLAVIGTERHESR  522
SECA1_ALK_metalliredigens  459  VLNAKHHEREAEIVAQAGRKGIITIATNMAGRGTDIILGGN----PEFLAKREMKKRGYADELIANATSHHETDDEELQAARKVYNDLLEKFKKETEQEHKDVIEAGGLHIIGTERHESR  574
SECA1_PEP_difficile        457  VLNAKHHDKAEEIIAQAGRLGAVTIATNMAGRGTDIVLGGNPDFLTKREMRRNGFKEEIVNRVDTPIEGIPVKGNEILFEAREEYEKLFEKFKQQTQEEQKQVVEAGGLAIIGTERHESR  576
SECA2_PEP_difficile        457  VLNAKQHDKEAEIISKAGKLDAITIATNMAGRGTDISLGA------GDKEEEQ------------------------------------------------EVKDLGGLYVIGTERHESR  522
SECA2_model_opened_conf    445  VLNAKQHDKEAEIISKAGKLDAITIATNMAGRGTDISLGA------GDKEEEQ------------------------------------------------EVKDLGGLYVIGTERHESR  510
SECA_E_coli_               478  VLNAKFHANEAAIVAQAGYPAAVTIATNMAGRGTDIVLGG------SWQAEVAALENPTAEQIEKIKADWQVRHDA--------------------------VLEAGGLHIIGTERHESR  565
SECA1_MYC_vanbaalenii      457  VLNAKYHEQEAGIVAEAGRLGAITVATNMAGRGTDIVLGG-----------------NVDYLLDRRLRQRGLDPIETPEEYEQGWHEELPHIKAEVAAEAKDVIAAGGLYVLGTERHESR  559
SECA1_MYC_ulcerans         456  VLNAKYHEQEAGIIAEAGRRGAITVATNMAGRGTDIVLGG-----------------NVDFLTDKRLRDNGLDPVETPDEYEQAWHQELPKVKEEAGDEATEVIKAGGLYVLGTERHESR  558
SECA1_MYC_paratuberculosi  456  VLNAKYHEQEAGIVAVAGRRGGVTVATHMAGRGTDIVLGG-----------------NVDFLTDQRLRERGLDPVETPDEYEAAWHEELPKVKAEAAAEAKEVIEAGGLYVLGTERHESR  558
SECA1_MYC_tuberculosis     459  VLNAKYHEQEATIIAVAGRRGGVTVATNMAGRGTDIVLGG-----------------NVDFLTDQRLRERGLDPVETPEEYEAAWHSELPIVKEEASKEAKEVIEAGGLYVLGTERHESR  561
SECA1_MYC_bovis            459  VLNAKYHEQEATIIAVAGRRGGVTVATNMAGRGTDIVLGG-----------------NVDFLTDQRLRERGLDPVETPEEYEAAWHSELPIVKEEASKEAKEVIEAGGLYVLGTERHESR  561
SECA1_MYC_leprae           459  VLNAKYHEQEAGIVAVAGRRGGVTVATNMAGRGTDIVLGG-----------------NVDFLTDQRLRERGLDPVETPDEYEQAWHSELPKVKEEAGQEAAEVIEAGGLYVLGTERHESR  561
SECA1_MYC_smegmatis        456  VLNAKYHEQEANIIAEAGRRGAVTVATNMAGRGTDIVLGG-----------------NVDFLADKRLRERGLDPVETPEEYEAAWHEVLPQVKAECAKEAEQVIEAGGLYVLGTERHESR  558
SECA1_COR_diphtheriae      458  VLNAKFHEQEAQIVAKAGLPGAVTVATNMAGRGTDIVLGG-----------------NADIIADINLRERGLNPVDTPEEYEAAWDAELARVKEKGAELAEKVREAGGLYVLGTERHESR  560
SECA1_COR_efficiens        458  VLNAKHHEQEAQIVAQAGLPGAVTVATNMAGRGTDIVLGG-----------------NPDILLDIKLRERGLDPFEDEEAYQVAWEEELPKMKQRCEERAEKVREAGGLYVLGTERHESR  560
SECA1_COR_glutamicum       458  VLNAKHHEQEAQIVAQAGLPGAVTVATNMAGRGTDIVLGG-----------------NPEILLDIKLRERGLDPFEDEESYQEAWDAELPAMKQRCEERGDKVREAGGLYVLGTERHESR  560
SECA1_COR_jeikeium         459  VLNAKYHEKEAEIVAQAGRLGAVTVATNMAGRGTDIVLGG-----------------NPDIIADINLRERGLDPVETPEEYEEAWDDEIEKVRKESKEEAEKVREVGGLYVLGTERHESR  561
SECA1_THE_fusca            459  VLNAKNHAREAAIVARAGKLGAVTVATNMAGRGTDIMLGG-----------------NPDFIAAEELQERGLSPLETPEEYEKAWPEALERAKKEVEAEHQKVVELGGLYVLGTERHESR  561
SECA2_MYC_vanbaalenii      457  VLNAKHLEQEAAIVAEGGRRGAVTVATDMAGRGTDIMLGG-----------------NVDFLTDKRLRSRGLHPTRSPEEYDAAWAEVRREVAAESRTEAREVVALGGLYVLGTERHESR  559
SECA_THE_maritima_         503  VLNAKYHEKEAEIVAKAGQKGMVTIATNMAGRGTDIKLGP-------------------------------------------------------------GVAELGGLCIIGTERHESR  561
SECA2_GEO_kaustophilus     458  LLNAKTEEEEARIIATAGQKGQVMIATNMAGRGTDILLGE-------------------------------------------------------------GVKELGGLHIIGTERHESH  516
SECA2_GEO_thermodenitrifi  459  LLNAKTVEQEAEVIARAGQRGRVTIATNIAGRGTDILLGE-------------------------------------------------------------GVNELGGLHVLGTERHESR  517
SECA2_BAC_anthracis        459  LLNAKSAEQEADLIATAGQKGQITIATNMAGRGTDILLGE-------------------------------------------------------------GVHELGGLHVIGTERHESR  517
SECA2_BAC_thuringiensis    459  LLNAKSAEQEADLIATAGQKGQITIATNMAGRGTDILLGE-------------------------------------------------------------GVHELGGLHVIGTERHESR  517
SECA2_BAC_cereus           459  LLNAKSAEQEADLIATAGQKGQITIATNMAGRGTDILLGE-------------------------------------------------------------GVHELGGLHVIGTERHESR  517
SECA2_LIS_welshimeri       452  VLNAKNHAQEAEIIAKAGKRGMVTLATNMAGRGTDIKLDP-------------------------------------------------------------DVHKLGGLAVIGTERHESR  510
SECA2_LIS_monocytogenes    452  VLNAKNHAQEAEIIAKAGKRGMVTLATNMAGRGTDIKLDP-------------------------------------------------------------DVHKLGGLAVIGTERHESR  510
SECA2_LIS_innocua          452  VLNAKNHAQEAEIIAKAGKRGMVTLATNMAGRGTDIKLDP-------------------------------------------------------------DVHKLGGLAVIGTERHESR  510
SECA2_ALK_metalliredigens  463  VLNAKNDWEEAKIIAEAGKYGAVTVSTNMAGRGIDIKLGG-----------------EKEL-------------------------------------DRKKVVTKGGLYVIGTSRHESS  528
SECA2_LAC_johnsonii        457  VLNAYNAAYEAQIIKNAGQKNAVTIATNMAGRGTDIKLGP-------------------------------------------------------------GVKELGGLAVIGTEML-PK  514
SECA2_STA_haemolyticus     462  LLIAQNVAKEAQMIAEAGQLGAVTVSTSMAGRGTDIKLGS-------------------------------------------------------------GVYELGGLAVIINEHMENS  520
SECA2_STA_aureus           462  LLIAQNVAKEAQMIAEAGQIGSMTVATSMAGRGTDIKLGE-------------------------------------------------------------GVEALGGLAVIIHEHMENS  520
SECA2_STA_epidermidis      462  LLIAQNVAKEAQMIAEAGQLSAVTVATSMAGRGTDIKLSK-------------------------------------------------------------EVHDIGGLAVIINEHMDNS  520
SECA2_STR_Agal             462  LLNAQSAVKEAQMIAEAGQKGAVTVATNMAGRGTDIKLGK-------------------------------------------------------------GVSELGGLAVIGTERMKSQ  520
SECA2_STR_Gordon           459  LLNANNVAREAQMIAESGQLGAVTVATSMAGRGTDIKLGP-------------------------------------------------------------GVADLGGLVVIGTERMENH  517
SECA2_STR_Pneum            458  VLNANNAAREAQIISESGQMGAVTVATSMAGRGTDIKLGK-------------------------------------------------------------GVAELGGLIVIGTERMESQ  516
SECA2_STR_Sanguin          459  LLNANNAAREAQIIAESGQKGAVTVATSMAGRGTDIKLGP-------------------------------------------------------------GVADLGGLVVIGTERMENQ  517
SECA2_COR_efficiens        456  VLNAKNDAEEARIIAEAGDIGRVTVSTQMAGRGTDVRLGG------PDESHYD------------------------------------------------QVVELGGLAVIGTARHRTA  521
SECA2_MYC_ulcerans         499  VLNAKNDAEEAAVIAEAGTLSRVTVSTQMAGRGTDIRLGG------SDEADHD------------------------------------------------QVAELGGLHVVGTGRHHTQ  564
SECA2_MYC_paratuberculosi  467  VLNAKNDAEEAQVIAEAGKFGVVTVSTQMAGRGTDIRLGG------SDEADHD------------------------------------------------RVAELGGLHVVGTGRHHTE  532
SECA2_MYC_tuberculosis     501  VLNAKNDAEEARVIAEAGKYGAVTVSTQMAGRGTDIRLGG------SDEADHD------------------------------------------------RVAELGGLHVVGTGRHHTE  566
SECA2_MYC_bovis            501  VLNAKNDAEEARVIAEAGKYGAVTVSTQMAGRGTDIRLGG------SDEADHD------------------------------------------------RVAELGGLHVVGTGRHHTE  566
SECA2_MYC_leprae           467  VLNAKNDAEEARFIAEAGKFGAVTVSTQMAGRGTDIRLGG------SDESDHD------------------------------------------------RVVELGGLHVVGTGRHHTE  532
SECA2_MYC_smegmatis        467  VLNAKNDAEEAAVIAEAGKLGAVTVSTQMAGRGTDIRLGG------SDVGDDD----AEKKK----------------------------------------VAELGGLHVVGTGRHHTE  536
SECA2_THE_fusca            456  VLNAKNDAEEAAVIAEAGTYGRITVSTQMAGRGTDIRLGG------SDMRDRD------------------------------------------------RVVKTGGLYVIGYGRYPSS  521
SECA2_COR_diphtheriae      456  VLNAKNDAEEAQIIAEAGDIGRVTVSTQMAGRGTDIRLGG------ANEKDRD------------------------------------------------AVVAKGGLAVIGSSRHRSS  521
SECA2_COR_glutamicum       456  VLNAKNDAEEAQIIAEAGDIGRVTVSTQMAGRGTDIRLGG------ADEADYD------------------------------------------------EVVKLGGLAVIGTARHRSQ  521
SECA2_COR_jeikeium         462  VLNAKNHEAEAAVVAEAGRPGRVTVSTQMAGRGTDIKLGG------TDEAHED------------------------------------------------EVVETGGLHVVGVGRFRSQ  527
Consensus_aa:                   lLNApp..pEApllptpAGp.G.VTltTpMAGRGTDI.LG..............................................................V.chGGLhVlGTpRhcop
Consensus_ss:                   ee    hhhhhhhhhh      eeeeehhh                                                                        hhh     e        h
```

```
Conservation:                          958 9869978989959 7 85585 955                              5          5   8   8     7      5        98  5   9  56
SECA1_GEO_thermodenitrifi  517  RIDNQLRGRSGRQGDPGVSQFYLSLEDELMRRFGSESLMAMMDRLGM-----DDSQPIQSKMVTRAVESAQKRVEGNNFDARKQLLQYDDVLREQREVIYRQRFEVLDADN--LRGIIEK  629
SECA1_GEO_kaustophilus     517  RIDNQLRGRSGRQGDPGVSQFYLSLEDELMRRFGSESLMAMMDRLGM-----DDSQPIQSKMVTRAVESAQKRVEGNNFDARKQLLQYDDVLREQREIIYRQRYEVLDSDN--LRGIIEK  629
SECA1_LAC_johnsonii        529  RIDNQLRGRSGRQGDPGVTRFYLSLEDDLMKRFGGDRVKLFLDRISDN----DDDKVIESRMITKQVESAQKRVEGNNYDTRKQTLQYDDVMRTQREIIYGERMQVISEDK-SLKPVLMP  643
SECA1_STR_parasanguinis    518  RIDNQLRGRSGRQGDPGESQFYLSLEDELMRRFGSERIKAVLDRFKLS----EEESVIRSNMFTRQVEGAQKRVEGNNYDTRKQVLQYDDVMREQREIIYAERYDVITANR-DLAPEIKA  632
SECA1_STR_gordonii         518  RIDNQLRGRSGRQGDPGESQFYLSLEDELMRRFGSERIKAVLDRFKLS----EEESVIKSKMFTRQVEAAQKRVEGNNYDTRKQVLQYDDVMREQREIIYAERHDVITANR-DLAPEIHA  632
SECA1_STR_pneumoniae       518  RIDNQLRGRSGRQGDPGESQFYLSLEDDLMKRFGSERLKGIFERLNMS------EEAIESRMLTRQVEAAQKRVEGNNYDTRKQVLQYDDVMREQREIIYAQRYDVITADR-DLAPEIQS  630
SECA1_STR_agalactiae       518  RIDNQLRGRSGRQGDPGESQFYLSLEDDLMRRFGTDRIKVVLERMNLA----EDDTVIKSKMLTRQVESAQRRVEGNNYDTRKQVLQYDDVMREQREIIYANRREVITAER-DLGPELKG  632
SECA1_LIS_monocytogenes    517  RIDNQLRGRSGRQGDPGVTQFYLSMEDELMRRFGSDNMKSMMERFGM-----AED-AIQSKMVSRAVESAQRRVEGNNFDSRKQVLQYDDVLRQQREVIYKQRYEVINAEN-SLREIIEQ  629
SECA1_LIS_innocua          517  RIDNQLRGRSGRQGDPGVTQFYLSMEDELMRRFGSDNMKSMMERFGM-----AED-AIQSKMVSRAVESAQRRVEGNNFDSRKQVLQYDDVLRQQREVIYKQRYEVINAEN-SLREIIEQ  629
SECA1_LIS_welshimeri_      517  RIDNQLRGRSGRQGDPGVTQFYLSMEDELMRRFGSDNMKSMMERFGM-----AED-AIQSKMVSRAVESAQRRVEGNNFDSRKQVLQYDDVLRQQREVIYKQRYEVINAEN-SLREIIEQ  629
SECA_BAC_subtilis          517  RIDNQLRGRSGRQGDPGITQFYLSMEDELMRRFGAERTMAMLDRFGM-----DDSTPIQSKMVSRAVESSQKRVEGNNFDSRKQLLQYDDVLRQQREVIYKQRFEVIDSEN--LREIVEN  629
SECA1_BAC_anthracis        516  RIDNQLRGRAGRQGDPGVTQFYLSMEDELMRRFGSDNMKAMMDRLGM-----DDSQPIESKMVSRAVESAQKRVEGNNYDARKQLLQYDDVLRQQREVIYKQRQEVMESEN--LRGIIEG  628
SECA1_BAC_thuringiensis    516  RIDNQLRGRAGRQGDPGVTQFYLSMEDELMRRFGSDNMKAMMDRLGM-----DDSQPIESKMVSRAVESAQKRVEGNNYDARKQLLQYDDVLRQQREVIYKQRQEVMESEN--LRGIIEG  628
SECA1_BAC_cereus           516  RIDNQLRGRAGRQGDPGVTQFYLSMEDELMRRFGSDNMKAMMDRLGM-----DDSQPIESKMVSRAVESAQKRVEGNNYDARKQLLQYDDVLRQQREVIYKQRQEVMESEN--LRGIIEG  628
SECA1_STA_epidermidis_     523  RIDDQLRGRSGRQGDRGDSRFYLSLQDELMVRFGSERLQKMMNRLGM-----DDSTPIESKMVSRAVESAQKRVEGNNFDARKRILEYDEVLRKQREIIYNERNEIIDSEE--SSQVVNA  635
SECA1_STA_haemolyticus     523  RIDDQLRGRSGRQGDRGDSRFYLSLQDELMVRFGSERLQKMMNRLGM-----DDSTPIESKMVSRAVESAQKRVEGNNFDARKRILEYDEVLRKQREIIYNERNNIIDSED--SSQLVNA  635
SECA1_STA_aureus           523  RIDDQLRGRSGRQGDKGDSRFYLSLQDELMIRFGSERLQKMMSRLGL-----DDSTPIESKMVSRAVESAQKRVEGNNFDARKRILEYDEVLRKQREIIYNERNSIIDEED--SSQVVDA  635
SECA1_ALK_metalliredigens  575  RIDNQLRGRAGRQGDPGSSKFYISLEDDLMRLFGGDKMLSIVEKMGL-----EDDEAIEHGMLSRSIENAQKKVEGRNFGIRKHVLQYDDVMNKQREVIYGERKKVLAGES--LKDHVLN  687
SECA1_PEP_difficile        577  RIDNQLRGRAGRQGDPGSSRFYIGLDDDLMRLFGSDRISGIVDKIGL-----EEDMPIEHRILSKSIEGAQKKVEGKNFGIRKHVLQYDDVMNKQREIIYAERKRVLEGED--LQEQIQS  689
SECA2_PEP_difficile        523  RIDNQLRGRSGRQGDPGTSRFFVSLEDDVIKLYGGKTIEKLMKRTSS-----NENTAIESKALTRAIERAQKGVEGKNFEIRKNVLKYDDTINEQRKVIYNERNKVLNDED--IQEDIQK  635
SECA2_model_opened_conf    511  RIDNQLRGRSGRQGDPGTSRFFVSLEDDVIKLYGGKTIEKLMKRTSS-----NENTAIESKALTRAIERAQKGVEGKNFEIRKNVLKYDDTINEQRKVIYNERNKVLNDED--IQEDIQK  623
SECA_E_coli_               566  RIDNQLRGRSGRQGDAGSSRFYLSMEDALMRIFASDRVSGMMRKLGM-----KPGEAIEHPWVTKAIANAQRKVESRNFDIRKQLLEYDDVANDQRRAIYSQRNELLDVSD--VSETINS  678
SECA1_MYC_vanbaalenii      560  RIDNQLRGRSGRQGDPGESRFYLSLADELMRRFNGATLETLLTRLNL-----PDDVPIEAKMVSRAIKSAQTQVEQQNFDIRKEVLKYDEVMNQQRKVVYAERRRILEGEN--LAGQAHQ  672
SECA1_MYC_ulcerans         559  RIDNQLRGRSGRQGDPGESRFYLSLGDELMRRFNGAALESLLTRLNL-----PDDVPIEAKMVTRAIKSAQTQVEQQNFEVRKNVLKYDEVMNQQRKVIYAERRRILEGEN--LQQQVKD  671
SECA1_MYC_paratuberculosi  559  RIDNQLRGRSGRQGDPGESRFYLSLGDELMRRFNGAALEAMLNRLNL-----PDDVPIEAKMVTRAIKSAQTQVEQQNFEVRKNVLKYDEVMNQQRKVIYAERRRILEGEN--LKEQALE  671
SECA1_MYC_tuberculosis     562  RIDNQLRGRSGRQGDPGESRFYLSLGDELMRRFNGAALETLLTRLNL-----PDDVPIEAKMVTRAIKSAQTQVEQQNFEVRKNVLKYDEVMNQQRKVIYAERRRILEGEN--LKDQALD  674
SECA1_MYC_bovis            562  RIDNQLRGRSGRQGDPGESRFYLSLGDELMRRFNGAALETLLTRLNL-----PDDVPIEAKMVTRAIKSAQTQVEQQNFEVRKNVLKYDEVMNQQRKVIYAERRRILEGEN--LKDQALD  674
SECA1_MYC_leprae           562  RIDNQLRGRSGRQGDPGESRFYLSLGDELMRRFHGATLEALLTRLNL-----PDDVPIEAKMVTRAIKSAQTQVEQQNFEVRKNVLKYDEVMNQQRKVIYAERRRILEGES--LQQQALG  674
SECA1_MYC_smegmatis        559  RIDNQLRGRSGRQGDPGESRFYLSLGDELMRRFNGATLETLLTRLNL-----PDDVPIEAKMVSRAIKSAQTQVEQQNFEVRKNVLKYDEVMNQQRKVIYAERRRILEGEN--LAEQAHK  671
SECA1_COR_diphtheriae      561  RIDNQLRGRAGRQGDPGTTRFYLSMRDDLMVRFVGQTMENMMNRLNV-----PDDVPIEAKMVTNSIKSAQTSVENQNFEMRKNVLKYDEVMNEQRKVIYTERREILESAD--IAADIQK  673
SECA1_COR_efficiens        561  RIDNQLRGRAGRQGDPGATRFYLSMRDDLMVRFVGPTMENMMNRLNV-----PDDVPIESKTVTNSIKGAQAQVENQNFEMRKNVLKYDEVMNEQRKVIYSERREILESSD--ISHYIKN  673
SECA1_COR_glutamicum       561  RIDNQLRGRSARQGDPGSTRFYLSMRDDLMVRFVGPTMENMMNRLNV-----PDDVPIESKTVTNSIKGAQAQVENQNFEMRKNVLKYDEVMNEQRKVIYSERREILESAD--ISRYIQN  673
SECA1_COR_jeikeium         562  RIDNQLRGRSARQGDPGETRFYLSMRDDLMVRFVGQTMEAMMTRLNI-----PDDEAIDSKMVTNAIKGAQSQVESANFEMRKNVLKYDEVMNEQRKVIYGERRQILEGED--VEKQIRS  674
SECA1_THE_fusca            562  RIDNQLRGRAGRQGDPGKSRFYLSLGDDLMRLFNGERVQMIMNRLNL-----PDDQPIEHKMVTKAIQSAQGQLEQQNFEIRKNVLKYDEVLNRQRQVIYAERRKVLEGAD--LREQVRS  674
SECA2_MYC_vanbaalenii      560  RIDNQLRGRSGRQGDPGETRFYVSLCDELMRRSATFDLEKLMSRLKM-----PEREPIEAKVVSRAIRNAQSQVEQANFDMRRSVVKYGQVLDQQRRIVYQARSRLLEGED--MQHQIFH  672
SECA_THE_maritima_         562  RIDNQLRGRAGRQGDPGESIFFLSLEDDLLRIFGSEQIGKVMNILKI-----EEGQPIQHPMLSKLIENIQKKVEGINFSIRKTLMEMDDVLDKQRRAVYSLRDQILLEKD--YDEYLKD  674
SECA2_GEO_kaustophilus     517  RIDMQLRGRAGRQGDPGSSQFIISLDDDLFRLYDQDELKKWKSKVET-----DETGRIVSPDPIKFVQKVQTTIENAHYSARLHLLKLDTVIDQQSKVIYHMRDRVLALKQAEVLSELLR  631
SECA2_GEO_thermodenitrifi  518  RIDNQLKGRAGRQGDPGSSQFFISLEDDMFRRFAAEETEKLKAKLKT-----DETGCILNNDIHEFVDKVQRIVEGMNFSVREYNLKLDDVMNEQRNVIYQIRDRVLEEN--DRVALVVP  630
SECA2_BAC_anthracis        518  RVDNQLKGRAGRQGDPGSSQFFLSLEDEMLKRFAQEEVEKLTKSLKT-----DETGLILTSKVHDFVNRTQLICEGSHFSMREYNLKLDDVINDQRNVIYKLRNNLLQEDT-NMIEIIIP  631
SECA2_BAC_thuringiensis    518  RVDNQLKGRAGRQGDPGSSQFFLSLEDEMLKRFAQEEVEKLTKSLKT-----DETGLILTAKVHDFVNRTQLICEGSHFSMREYNLKLDDVINDQRNVIYKLRNNLLQEDT-NMIEIIIP  631
SECA2_BAC_cereus           518  RVDNQLKGRAGRQGDPGSSQFFLSLEDEMLKRFAQEEIEKLTKSLKT-----DETGLILTAKVHDFVNRTQLICEGSHFSMREYNLKLDDVINDQRNVIYKLRNNLLQEDT-NMIEIIIP  631
SECA2_LIS_welshimeri       511  RIDLQLMGRSGRRGDPGFSKFMISLEDDLLEQFESKSWEKLSVKLKRK--APRDGKPVNSSKIHAVVVNAQKRLEGANYDIRKDLLSYDEVIDLQRKMVYKERDLLLERNK--LGVSSEK  626
SECA2_LIS_monocytogenes    511  RIDLQLMGRSGRRGDPGFSKFMISLEDDLLEQFESKSWEKLSAKLKRK--APRDGKPVNSRKIHAVVVDAQKRLEGANYDIRKDLLSYDEVIDLQRKMVYKERDLLLERNK--LGVSSEK  626
SECA2_LIS_innocua          511  RIDLQLMGRSGRRGDPGFSKFMISLEDDLLEQFESKSWEKLSTKLKRK--APRDGKPVNSRKIHSVIVDAQKRLEGANYDIRKDLLSYDEVIDLQRKMVYKERDQLLERNK--LGVSSEK  626
SECA2_ALK_metalliredigens  529  RIDNQLRGRAGRQGDPGESKFFISLEDDLIKKYEIDKMIPQEY-YTQ-----KQSESLDNTMINRKINSGQRIVEGYNSDIRKQLWEYSYVIEEQRRIIHQKRHEILVDRE--PLSLLSN  640
SECA2_LAC_johnsonii        515  RVELQLAGRAGRQGDPGSSQFLISLEDSFISSNNTPRQKKYYRKLMKKKSKGKDITLLSGPRIRFSLLMLRTRKEDLNELMRSQTNKMEIILSLQRKNFYTRRDKIMKTDD--LQEDVDR  632
SECA2_STA_haemolyticus     521  RVDRQLRGRSGRQGDPGVSQIYVSLDDYIVKRWSNSKLAENKKLKDVDP-DKLQDSPFFRRRVRGIVSKAQRVSEETSMMAREMANEFEKSIGIQRDRVYEERNRILETSD--FSAFDFD  637
SECA2_STA_aureus           521  RVDRQLRGRSGRQGDPGSSCIYISLDDYLVKRWSDSNLAENNQLYSLDA-QRLSQSNLFNRKVKQIVVKAQRISEEQGVKAREMANEFEKSISIQRDLVYEERNRVLEIDD--AENRDFK  637
SECA2_STA_epidermidis      521  RVDRQLRGRSGRQGDPGYSQIFVSLDDDLVKRWSNSNLAENKNLQTMDA-SKLESSALFKKRVKSIVNKAQRVSEETAMKNREMANEFEKSISVQRDKIYAERNHILEASD--FDDFNFE  637
SECA2_STR_Agal             521  RMDLQLRGRSGRQGDIGFSQFFVSFEDDLMIESGPKWAQAYFRKNRDKV-NPEKPKALGQRRFQKLFQQTQEASDGKGESARSQTIEFDSSVQLQREYVYRERNALINGES--GHFSPRQ  637
SECA2_STR_Gordon           518  RIDLQIRGRSGRQGDPGISKFFISLEDDLLKKWGPDWIKNFYRDYSPED-FKNNPILLKQRRFKKLVSKAQKASEGNAKLSRRLTLEYAQSMKIQRELTYAERNRLLEDTN-EMEEEINR  635
SECA2_STR_Pneum            517  RIDLQIRGRSGRQGDPGMSKFFVSLEDDVIKKFGPSWVHKKYKDYQVQD-MTQ-PEVLKGRKYRKLVEKAQHASDSAGRSARRQTLEYAESMNIQRDIVYKERNRLIDGSR-DLEDVVVD  633
SECA2_STR_Sanguin          518  RIDLQIRGRSGRQGDPGISKFFISLEDDLLRKWGPDWLKKLYKDYSTEE-VQQHPVQLGQRRFRRLVAKAQRASESSAKMSRRMTLEYAQCMKIQREITYAERNRLIQAEE-RIDEEISR  635
SECA2_COR_efficiens        522  RLDNQLRGRAGRQGDPGLSLFFVSLEDDVVVTGGAGESVTAQPD---------ATGLIDSNRVRDWVAHCQRVTEGQLLEIHSQTWKYNKLLADQRVIIDERRARLLDTDL--AWRELSE  630
SECA2_MYC_ulcerans         565  RLDNQLRGRAGRQGDPGSSVFFSSWEDDVVAANLDGNKLPMETD---------EDGQIVSAKAAGLLDHAQRVAEGRMLDVHANTWRYNQLIAQQRAIIVDRRNTLLRTAT--AREELAD  673
SECA2_MYC_paratuberculosi  533  RLDNQLRGRAGRQGDPGSSVFFSSWEDDVVAANLDRNKLPMETDP------ETGDGRIVSPKAAGLLDHAQRVAEGRMLDVHANTWRYNQLIAQQRAIIVDRRNTLLRTAT--AREELAE  644
SECA2_MYC_tuberculosis     567  RLDNQLRGRAGRQGDPGSSVFFSSWEDDVVAANLDHNKLPMATD---------ENGRIVSPRTGSLLDHAQRVAEGRLLDVHANTWRYNQLIAQQRAIIVERRNTLLRTVT--AREELAE  675
SECA2_MYC_bovis            567  RLDNQLRGRAGRQGDPGSSVFFSSWEDDVVAANLDHNKLPMATD---------ENGRIVSPRTGSLLDHAQRVAEGRLLDVHANTWRYNQLIAQQRAIIVERRNTLLRTVT--AREELAE  675
SECA2_MYC_leprae           533  RLDNQLRGRAGRQGDPGSSVFFSSWEDDVIAANLDRNKLPMQTD---------EDGRIISLKTTGLLDHAQRVAEGRLLDVHANTWRYNQLIAQQRAIIVDRRNALLSSAT--AREELAE  641
SECA2_MYC_smegmatis        537  RLDNQLRGRAGRQGDPGSSVFFSSWEDDVVAAHLERSKLPMETDP------DAGDGRIIAPRAASLLDHAQRVAEGRLLDVHANTWRYNQLIAQQRAIIVERRETLLRTDT--AREELKE  648
SECA2_THE_fusca            522  RLDDQLRGRAGRQGDPGGSVFYVSVEDDLITTNLPEAKGYRVSS---------ADGEITDPAWKEMVNHAQRIAEGQLLELHRNTWRYNQIIDVQRSVVLEQRRAVLHEDL--GSRQLAV  630
SECA2_COR_diphtheriae      522  RLDNQLRGRAGRQGDPGLSLFFVSLEDDVVVVGGAGEEIKALPD---------ADGRIDSKRITDFVAHCQRVTEGQLLEIHSQTWKYNKLLADQRVIIDERRARLLDTDQ--AWVELSE  630
SECA2_COR_glutamicum       522  RLDNQLRGRAGRQGDPGLSLFFVSLDDDVVVSGGSGESVSAQPD---------ATGLIDSDRIRDWVGHCQRVTEGQLLEIHSQSWNYNKLLADQRVIIDERRERLLDTAL--AWEELAQ  630
SECA2_COR_jeikeium         528  RLDNQLRGRAGRQGDPGSSLFFVSLEDDVVAVGGAGEELQAQPE---------EDGLLPQKKVQQFVDHCQRVTEGQMLDIHATTWKYNKLIKDQRDIVNDRRDTLLDTAA--AWDDLSY  636
Consensus_aa:                   RlD.QLRGRtGRQGDPG.S.F@lSh-D-lh....s.pb..h.pch........ps..l.p..hpphl.ptQp.sEs..hphRpphhp@sphhp.QRphl@.pRppllp.pp..h...h..
Consensus_ss:                   hhhhhh                eeeeee hhhhhhhhhhhhhhhhhhhh                  hhhhhhhhhhhhhhhhhhhhhhhhhhhhhhhhhhhhhhhhhhhhhhhh      hhhhhhh
```

HWD domain

```
Conservation:                                                                                                               6   5  58  9  55  5  65
SECA1_GEO_thermodenitrifi  630  MIRSVIERVVNTYTPKEDLPEEWNLKG-VVDYLNAYLLPEGDVT--EGDLR-------GKEPEEMIELIWAKVKARYDEKETQI----PPEQMREFERVVVLRAVDMKWMNHIDAMEQLR  735
SECA1_GEO_kaustophilus     630  MIHSVIERVVNAHTPKEEVPEEWNLKG-LVEYLNAHLLPEGDVT--EADLR-------GKEPEEMIELIWAKVKARYDEKEAQI----PPEQMREFERVVVLRAVDMKWMHHIDAMEQLR  735
SECA1_LAC_johnsonii        644  MIKRTIDHQIDMYTQGDK--KDWRNDQ-IRDFISSAITDEETTK--KLNMK-------HLSAEELKKRLYQIAEDNYAEKEKQL---ADPEQMLEFEKVVILRVVDERWTDHIDAMDQLR  748
SECA1_STR_parasanguinis    633  MIKRTIKRIVEGASHSSK---EERVEA-ILNFAKYNLVPEDTIS--ESDIE-------GKSDKEVIDYLYARAEEIYASQVAKL---RDEESVQEFQKVLILRVVDSKWTDHIDALDQLR  736
SECA1_STR_gordonii         633  MIKRTIDRFVDGNSRAPQ---EEKLDS-ILYFAKYNLVPEESIS--LSDLQ-------GLSDEEIKANLYERALEVYNSQIAKL---RDEEAVREFQKVLILRVVDNKWTDHIDALDQLR  736
SECA1_STR_pneumoniae       631  MIKRTIERVVDGHARAKQ---DEKLEA-ILNFAKYNLLPEDSIT--MEDLS-------GLSDKAIKEELFQRSLKVYDSQVSKL---RDEEAVKEFQKVLILRVVDNKWTDHIDALDQLR  734
SECA1_STR_agalactiae       633  MIKRTIKRAVDAHSRSDK---NTAAEA-IVNFARSALLDEEAIT--VSELR-------GLKEAEIKELLYERALAVYEQQIAKL---KDPEAIIEFQKVLILMVVDNQWTEHIDALDQLR  736
SECA1_LIS_monocytogenes    630  MIQRTVNFIVSSNASSHEPEEAWNLQG-IIDYVDANLLPEGTIT--LEDLQ-------NRTSEDIQNLILDKIKAAYDEKETLL----PPEEFNEFEKVVLLRVVDTKWVDHIDAMDHLR  735
SECA1_LIS_innocua          630  MIQRTVNFIVSSNASSHEPEEAWNLQG-IIDYVDANLLPEGTIT--LEDLQ-------NRTSEDIQNLILDKIKAAYDEKETLL----PPEEFNEFEKVVLLRVVDTKWVDHIDAMDHLR  735
SECA1_LIS_welshimeri_      630  MIQRTVNYIVSSNASSREPEEDWNLQG-IIDYVDANLLPEGTVT--LEDIQ-------NRSSEDIQNLILDKIKVAYDEKETLL----PPEEFNEFEKVVLLRVVDTKWVDHIDAMDHLR  735
SECA_BAC_subtilis          630  MIKSSLERAIAAYTPREELPEEWKLDG-LVDLINTTYLDEGALE--KSDIF-------GKEPDEMLELIMDRIITKYNEKEEQF----GKEQMREFEKVIVLRAVDSKWMDHIDAMDQLR  735
SECA1_BAC_anthracis        629  MMKSTVERAVALHT-QEEIEEDWNIKG-LVDYLNTNLLQEGDVK--EEELR-------RLAPEEMSEPIIAKLIERYNDKEKLM----PEEQMREFEKVVVFRVVDTKWTEHIDAMDHLR  733
SECA1_BAC_thuringiensis    629  MMKSTVERAVALHT-QEEIEEDWNIKG-LVDYLNTNLLQEGDVK--EEELR-------RLAPEEMSEPIIAKLIERYNDKEKLM----PEEQMREFEKVVVFRVVDTKWTEHIDAMDHLR  733
SECA1_BAC_cereus           629  MMKSTVERAVALHT-QEEIEEDWNIKG-LVDYLNTNLLQEGDVK--EEELR-------RLAPEEMSEPIIAKLIERYNDKEKLM----PEEQMREFEKVVVFRVVDTKWTEHIDAMDHLR  733
SECA1_STA_epidermidis_     636  MLRSTLQRAINHFINEED--DNPDYTP-FINYVNDVFLQEGDLQ--DTEIK-------GKDSEDIFEIVWSKIEKAYAQQQETL-----GDQMSEFERMILLRSIDTHWTDHIDTMDQLR  738
SECA1_STA_haemolyticus     636  MLRSTLQRGVTYHISEED--DNPDYAP-FINYVNDVFLQEGDLK--EEEIN-------GKDSEDIFEVVWSKIEKVYESQKAKI-----GDQMAEFERMILLRSIDTHWTDHIDTMDQLR  738
SECA1_STA_aureus           636  MLRSTLQRSINYYINTAD--DEPEYQP-FIDYINDIFLQEGDIT--EDDIK-------GKDAEDIFEVVWAKIEAAYQSQKDIL-----EEQMNEFERMILLRSIDSHWTDHIDTMDQLR  738
SECA1_ALK_metalliredigens  688  MARNIINEAVAIYTADAKYPEEWDLVG-LGEYLAGIYMQRATLS--FDNIE-------ELTVETLQEQIYETSEKLYEAKEEEI----EAERMRELERIIVLQVIDTKWMDHIDAMDQLR  793
SECA1_PEP_difficile        690  MTHSIIEEAVTLYTQDKG----FDEEG-FKEHMYNLFLPKGSIE--IPEIE-------KLNPVEITEKVYEIAMKIYTSKEEQV----GYERMREVERVILLQAVDNHWIDHIDAMDQLR  791
SECA2_PEP_difficile        636  MVKDIIQEAGETYLIGRK----RDYYG-YFKHLYSTFMPADTLL--IPGVD-------KKSVQEIIDSTYEISKRVYDLKKMML----GIDKVAELEKTVLLKVVDQYWIDHIDAMEQLK  737
SECA2_model_opened_conf    624  MVKDIIQEAGETYLIGRK----RDYYG-YFKHLYSTFMPADTLL--IPGVD-------KKSVQEIIDSTYEISKRVYDLKKMML----GIDKVAELEKTVLLKVVDQYWIDHIDAMEQLK  725
SECA_E_coli_               679  IREDVFKATIDAYIPPQSLEEMWDIPG-LQERLKNDFDLDLPIAEWLDKEP-------ELHEETLRERILAQSIEVYQRKEEVV----GAEMMRHFEKGVMLQTLDSLWKEHLAAMDYLR  786
SECA1_MYC_vanbaalenii      673  ILVDVITAYVDGATAEG-YSEDWDLEK-LWEGLRQLYPVGIDHH--DLIDSDAVGEPGELTREELLQALIADAERAYAAREAEIEEIAGEGAMRQLERNVLLNVLDRKWREHLYEMDYLR  788
SECA1_MYC_ulcerans         672  MLTDVITAYVDGATVEG-YAEDWDLDA-LWTALKTLYPVGIKTD--TLMRRDQDSDRDDLTRDELLQALLQDADQAYAAREAELEELAGEGAMRQLERNVLLNVIDRKWREHLYEMDYLK  787
SECA1_MYC_paratuberculosi  672  MVRDVVTAYVNGATAEG-YAEDWDLDA-LWTALKTLYPVGIDYA--TLTRRDADGGFDDLTREELLEALLKDAERAYATREAELEEIAGEGAMRQLERNVLLNVIDRKWREHLYEMDYLK  787
SECA1_MYC_tuberculosis     675  MVRDVITAYVDGATGEG-YAEDWDLDA-LWTALKTLYPVGITAD--SLTRKDHEFERDDLTREELLEALLKDAERAYAAREAELEEIAGEGAMRQLERNVLLNVIDRKWREHLYEMDYLK  790
SECA1_MYC_bovis            675  MVRDVITAYVDGATGEG-YAEDWDLDA-LWTALKTLYPVGITAD--SLTRKDHEFERDDLTREELLEALLKDAERAYAAREAELEEIAGEGAMRQLERNVLLNVIDRKWREHLYEMDYLK  790
SECA1_MYC_leprae           675  MVRDVITAYIDGATTDS-YVEDWDLDA-LWSALGTLYPVGIKHE--SLTHADEDSECDELSRDELLEALLIDAQHAYAAREAELAELAGEGAMRQLERNVLLNVVDRKWREHLYEMDYLK  790
SECA1_MYC_smegmatis        672  MLVDVITAYVDGATAEG-YAEDWDLET-LWTALKTLYPVGIDHR--DLIDSDAVGEPGELTREELLDALIKDAERAYAEREKQIEAIAGEGAMRQLERNVLLNVIDRKWREHLYEMDYLK  787
SECA1_COR_diphtheriae      674  MIDDTIGAYVDAATATG-YVEDWDLET-LWNALESLYGPSFSAQ--ELIDGDSYGESGELSASDLRKAVLEDAHKQYAELEENVTAIGGEAQMRNIERMVILPVIDTKWREHLYEMDYLK  789
SECA1_COR_efficiens        674  MVDETISAYVAAATANG-YVEDWDLDK-LWNALEALYGPTFTWQ--SLVDGSEYGAPGELSAEDLRTALLEDARAEYAKLEEAVTALGGEAQMRNIERMVLMPVIDQKWREHLYEMDYLK  789
SECA1_COR_glutamicum       674  MIEETVSAYVDGATANG-YVEDWDLDK-LWNALEALYDPSINWT--DLVEGSEYGKPGELSAEDLRTALVNDAHAEYAKLEEAVSAIGGEAQIRNIERMVLMPVIDTKWREHLYEMDYLK  789
SECA1_COR_jeikeium         675  MLKDTIEAYVDGATAEG-YVEDWDLDT-LWNALDSLYGPTFTHE--ELVEGDEYGRPGELSSSQLLDALLEDANREYDELEEKVSEVAGEEQMRGMERAALLNVVDQKWREHLYEMDYLK  790
SECA1_THE_fusca            675  MIDDVLDSYVRSATAEG-DPEDWDLEH-LWTAFSQIFPVSFTAD--QLIEENGG-DISVLTPDIISQRVREDAHEVYDRREAEI----GEETMREVERQVILQVMDRKWREHLYEMDYLQ  785
SECA2_MYC_vanbaalenii      673  MIGDVVTAYVNECTAGR-RTADWDLET-LRAALSTLYPV--------VWQPDPRPHMRGLTRSVLRHEVIADARRALVRRKAAIEARSGLRVMRELERAILLDCLDSKWRAHLYEMDYLA  782
SECA_THE_maritima_         675  IFEDVVSTRVEEFC----SGKNWDIES-LKNSLSFFPAGLFDL----DEKQ-------FSSSEELHDYLFNRLWEEYQRKKQEI-----GEDYRKVIRFLMLRIIDDHWRRYLEEVEHVK  773
SECA2_GEO_kaustophilus     632  HIHRHITQTIDRYCPENVFFEEWNIEG-LHNELRRAFFRFAYP---IDDLR-------HKQKEEIAQLVWDEYKSLEAALADLP----CDEEQTMRLKHLMVETIDAHWIRHLNQLNLLK  736
SECA2_GEO_thermodenitrifi  631  MIRSACDRIVDAYALSEQIPEEWDVRR-MTEELNRI--VYRTPI--TFDQP-------PADLEDVKRKVAEAVESYVALLEKKK----AHTQLQTLLKSVMLTVIDDYWMRHLDQMALLK  734
SECA2_BAC_anthracis        632  MIDHAVEAISKQYLVEGMLPEEWDFAS-LTASLNEILSVENMPS--LSANN-------VHSPEDLQSVLKETLSLYKERVNELD----SNTDLQQSLRYVALHFLDQNWVNHLDAMTHLK  737
SECA2_BAC_thuringiensis    632  MIDHAVEAISKQYLVEGMLPEEWDFAS-LTASLNEILSVENMPS--LSANN-------VHSPEDLQSVLKETLSLYKERVNELD----SHTDLQQSLRYVALHFLDQNWVNHLDAMTHLK  737
SECA2_BAC_cereus           632  MIDHAVEAISKQYLVEGMLPEEWDFAS-LTASLNEILSVENMPS--LSANN-------VHSPEDLQSVLKETLSLYKERVNELD----SNTDLQQSLRYVALHFLDQNWVNHLDAMTHLK  737
SECA2_LIS_welshimeri       627  ILREVAEYAFIHPV----DIEQEKLEK-YYARQKELLGGTKFPV-SFDEVS-------LMEPAEVVEKI----VTWHKKERDKF----PIETITAIEKEVYLNLMDQMWVMHLDAMVQLR  725
SECA2_LIS_monocytogenes    627  ILREVAEYSFIHPS----DIPEEELEI-YYSRQKELLGGTKFPI-SFDQVT-------LMEPREVVEEI----VSWHKKERNKF----PAETIAAIEREVYLNLMDQMWVMHLDAMVQLR  725
SECA2_LIS_innocua          627  ILREVAEYSFIHPL----ELEEEELEK-YYSRQKELLGGTKFPI-SFDQVT-------LMDPVEVVEEI----VAWHKKERNKF----PVETITAIEKEVYLNLMDQMWVMHLDAMVQLR  725
SECA2_ALK_metalliredigens  641  KCPVLYDRYLDLVG----------------------------------------------------------------------------KDVLYKVEKQITLFHINKCWAEYLEYVSYIR  685
SECA2_LAC_johnsonii        633  IIDNALNLYLEKQD----LSNKSDLKY-FINQHVTYQQVNVPDN--------------LKTKKAIKDFLKELAYKILDEKKHVL---INKKQANDFYQQVIISSMDGNWIDQVDRIEKIK  730
SECA2_STA_haemolyticus     638  SLARDVFDYDLRTKH---IHNKDDIINYIYEQLSFSFKDDA------ISQQ-------IQTREQTIDYLVQQFNKQLKENMKIA---NNDYFKLRFFQKAILKAIDVEWINQVDQLQQLK  738
SECA2_STA_aureus           638  ALAKDVFEMFVNEEK---VLTKSRVVEYIYQNLSFQFNKDV------ACVN-------FKDKQAVVTFLLEQFEKQLALNRKNM---QSAYYYNIFVQKVFLKAIDSCWLEQVDYLQQLK  738
SECA2_STA_epidermidis      638  QLARDVFTKDVKNLD---LSSERALVNYIYENLSFVFDEDV------SNIN-------MQNDEEIIQFLIQQFTQQFNNRLEVA---ADSYLKLRFIQKSILKAIDSEWIEQVDNLQQLK  738
SECA2_STR_Agal             638  IIDTVISSFIAYLDGE--VEKEELIFE-INRFIFDNMSYNLQ-----GISK-------EMSLEEIKNYLFKIADEILREKHNLL-----GDSFGDFERTAALKAIDEAWIEEVDYLQQLR  737
SECA2_STR_Gordon           636  VIEYVIKQVAYEQS----FENRADFYR-FILHHFSNRAERIP-----QEFD-------IHSPKEVSHLLQAIAEQELLAKKSYL---KSDKLYSQFQRLAILKAIDENWVEQVDYLQQLK  735
SECA2_STR_Pneum            634  IIERYTEEVAADH-----YASRELLFH-FIVTNISFHVKEVP-----DYID-------VTDKTAVRSFMKQVIDKELSEKKELL---NQHDLYEQFLRLSLLKAIDDNWVEQVDYLQQLS  732
SECA2_STR_Sanguin          636  VLSQVIHQAAYEQS----YETRADLYR-FILDHFSYHAERIP-----YDFD-------IYSPEKIAELLQDIAEQELQAKKAYL---KSDKLFTHFQRVSVLKAIDENWVEQVDYLQQLK  735
SECA2_COR_efficiens        631  RAQDRAAGLEG-V-----------------------------------------------------------------------DREVLEQAARDIMLYHLDLNWSEHLALMDDVR  674
SECA2_MYC_ulcerans         674  LAPKRYKELSETV-----------------------------------------------------------------------SEHRLEKICRMIMLYHLDRGWADHLAYLADIR  718
SECA2_MYC_paratuberculosi  645  LAPKRYRELADEI-----------------------------------------------------------------------PEERLETICRHIMLYHLDRGWADHLAYLADIR  689
SECA2_MYC_tuberculosis     676  LAPKRYEELSDKV-----------------------------------------------------------------------SEERLETICRQIMLYHLDRGWADHLAYLADIR  720
SECA2_MYC_bovis            676  LAPKRYEELSDKV-----------------------------------------------------------------------SEERLETICRQIMLYHLDRGWADHLAYLADIR  720
SECA2_MYC_leprae           642  LAPKRYEELAQALPKEE-----------------------------------------------------------------------AEERLETICRLIMLYHLDRGWADHLAYLADIR  690
SECA2_MYC_smegmatis        649  RSPERYAKLAEEL-GED-----------------------------------------------------------------------AEERLEKICRLIMLYHLDRGWCEHLAFLADIR  696
SECA2_THE_fusca            631  DCPETYQRLVEEV-----------------------------------------------------------------------GEEEVARAARLVTLYHLDRGWADHNAFLADLR  675
SECA2_COR_diphtheriae      631  AVPEKAHKLSDKL-----------------------------------------------------------------------DPAILVQAAREVMLYHLDRCWSDHLALMDHVR  675
SECA2_COR_glutamicum       631  HAPARAAELEDL------------------------------------------------------------------------DQSVREQAARDIMLYHLDYNWSEHLALMDDVR  674
SECA2_COR_jeikeium         637  HNVDRAAELKKQGI----------------------------------------------------------------------SEEVLEQAAREIMLFHLDNEWSEHLAYLDDVR  682
Consensus_aa:                   hh.p.hp.h...........c.ph...hh..h...h............p........s.ppl.p.lb..h.p.h..pp..h....s.p.h.ph.+.lhL.hlDp.W.cpls.hp.l+
Consensus_ss:                   hhhhhhhhhhhh              hhh hhhhhhhh              hhh            hhhhhhhhhhhhhhhhhhhhhh       hhhhhhhhhhhhhhhhhhhhhhhhhhhhhhhh
```

```
                                                2HF loop
Conservation:                          5     5   6   6   5   7  5
SECA1_GEO_thermodenitrifi   736   QGIHLRAYGQVDPLREYQMEGYAMFENMIAAIEEEVATYIMKAEIH-----------HNLERQEVAK--GEA------------------------------------------------   794
SECA1_GEO_kaustophilus      736   QGIHLRAYGQVDPLREYQMEGYAMFEEMIAAIEEEVATYIMKAEIH-----------HNLERQEVAK--GEA------------------------------------------------   794
SECA1_LAC_johnsonii         749   QSISLRGYGQLNPLVEYQESGYRMFEEMISNIEFDATRLFMKAQIR-----------QNISR----------------------------------------------------------   799
SECA1_STR_parasanguinis     737   NAVGLRGYAQNNPVVEYQSESFRMFNDMIGSIEFDVTRLMMKAQIH------------EQERPRTEH---SI------------------------------------------------   793
SECA1_STR_gordonii          737   NAVGLRGYAQNNPVVEYQSESFRMFNDMIGSIEFDVTRLMMKAQIH------------EQERPRTEH---NI------------------------------------------------   793
SECA1_STR_pneumoniae        735   NAVGLRGYAQNNPVVEYQAEGFRMFNDMIGSIEFDVTRLMMKAQIH------------EQERPQAER---HI------------------------------------------------   791
SECA1_STR_agalactiae        737   NSVGLRGYAQNNPIVEYQSEGFRMFQDMIGSIEFDVTRTLMKAQIH------------EQERERASQ---HA------------------------------------------------   793
SECA1_LIS_monocytogenes     736   DGIHLRAYGQIDPLREYQSEGFEMFEAMVSSIDEDVARYIMKAEIR-----------QNLEREQVAK--GEA------------------------------------------------   794
SECA1_LIS_innocua           736   DGIHLRAYGQIDPLREYQSEGFEMFEAMVSSIDEDVARYIMKAEIR-----------QNLEREQVAK--GEA------------------------------------------------   794
SECA1_LIS_welshimeri_       736   DGIHLRAYGQIDPLREYQSEGFEMFEAMVSSIDEDVARYIMKAEIR-----------QNLEREQVAK--GEA------------------------------------------------   794
SECA_BAC_subtilis           736   QGIHLRAYAQTNPLREYQMEGFAMFEHMIESIEDEVAKFVMKAEIE-----------NNLEREEVVQ--GQTTA----------------------------------------------   796
SECA1_BAC_anthracis         734   EGIHLRAYGQIDPLREYQMEGFAMFESMIASIEEEISRYIMKAEIE-----------QNLERQEVVQ--GEA------------------------------------------------   792
SECA1_BAC_thuringiensis     734   EGIHLRAYGQIDPLREYQMEGFAMFESMIASIEEEISRYIMKAEIE-----------QNLERQEVVQ--GEA------------------------------------------------   792
SECA1_BAC_cereus            734   EGIHLRAYGQIDPLREYQMEGFAMFESMIASIEEEISRYIMKAEIE-----------QNLERQEVVQ--GEA------------------------------------------------   792
SECA1_STA_epidermidis_      739   QGIHLRSYAQQNPLRDYQNEGHELFDIMMQNIEEDTCKYILKSVVQF---------EDDVEREKSKS--FGEAKH---------------------------------------------   802
SECA1_STA_haemolyticus      739   QGIHLRSYAQQNPLRDYQNEGHELFDMMMQNIEEDTSKFILKSVIQV---------DENIEREKTTD--FGTAQH---------------------------------------------   802
SECA1_STA_aureus            739   QGIHLRSYAQQNPLRDYQNEGHELFDIMMQNIEEDTCKFILKSVVQV---------EDNIEREKTTE--FGEAKH---------------------------------------------   802
SECA1_ALK_metalliredigens   794   QGIGLRAIGQIDPVRAYQVEGFDMFNAMINSIQEDTVKYLFNVEPQ-----------AKVERKQVAK----P------------------------------------------------   850
SECA1_PEP_difficile         792   QGIGLRAVGQQDPVIAYKMEGFDMFDEMNKHIKEDTVRYLFNITIE-----------TPVERKAVVD--VEN------------------------------------------------   850
SECA2_PEP_difficile         738   QYIGLKSYAQKDPFKEYALEGYDMFEALNKNIREATVQYLYKFN----------------------------------------------------------------------------   781
SECA2_model_opened_conf     726   QYIGLKSYAQKDPFKEYALEGYDMFEALNKNIREATVQYLYKFN----------------------------------------------------------------------------   769
SECA_E_coli_                787   QGIHLRGYAQKDPKQEYKRESFSMFAAMLESLKYEVISTLSKVQVRMPEEVEELEQQRRMEAERLAQ--MQQ------------------------------------------------   856
SECA1_MYC_vanbaalenii       789   EGIGLRGLAQQRPEVEYAREGYDMFIAMLDGMKEESVGFLFNVQVE-----------RAPSAPTVAA--QAAPAGLAAFAAAAAEQAQAQTGGVATKERPA-------------------   876
SECA1_MYC_ulcerans          788   EGIGLRAMAQRDPLVEYQREGYDMFMAMLDGVKEESVGFLFNVSVE-----------AVPA-PQVEVAPVAEPEDLAEFATAAAAAAQEGGAGRKNAAAREE------------------   877
SECA1_MYC_paratuberculosi   788   EGIGLRAMAQRDPLVEYQREGYDMFMAMLDGMKEESVGFLFNVTVE-----------AVPA-PQVAP--VQTPEGLAELGAPAEQGGTATAARDEAPT----------------------   871
SECA1_MYC_tuberculosis      791   EGIGLRAMAQRDPLVEYQREGYDMFMAMLDGMKEESVGFLFNVTVE-----------AVPA-PPVAP--AAEPAELAEFAAAAAAAAQQRSAVDGGARERA-------------------   877
SECA1_MYC_bovis             791   EGIGLRAMAQRDPLVEYQREGYDMFMAMLDGMKEESVGFLFNVTVE-----------AVPA-PPVAP--AAEPAELAEFAAAAAAAAQQRSAVDGGARERA-------------------   877
SECA1_MYC_leprae            791   EGIGLRAMAQRDPLVEYQREGYDMFMAMLDGVKEESVGFLFNLAVE-----------AVPA-SHVAP--VEIPEGFTGLVADVEAIRPREEASSAL------------------------   872
SECA1_MYC_smegmatis         788   EGIGLRAMAQRDPLVEYQREGYDMFVGMLEALKEESVGFLFNVQVE-----------AAPQQPQVAP--QAPPPTLSEFAAAAAAKASDSAAKPDSGSVATKERA---------------   879
SECA1_COR_diphtheriae       790   EGIGLRAMAQRDPLVEYQKEGGDMFNAMKDAVKEETVRQLFLLRKQ-----------FAVAN-------EQPAETEEGTVEA--------------------------------------   853
SECA1_COR_efficiens         790   EGIGLRAMAQRDPLVEYQKEGGDMFNAMKEAIKEETVRQLFLMRKQ-----------FVKQD-------EEANA----------------------------------------------   845
SECA1_COR_glutamicum        790   EGIGLRAMAQRDPLVEYQKEGGDMFNGMKDGIKEETVRQLFLLRKQ-----------FIKQD-------AEVAD----------------------------------------------   845
SECA1_COR_jeikeium          791   EGIGLRAMAQRDPLVEYQREGGDMFNRMKDGIKEETVRQLFLVRNQ-----------LKQA-GQVHV--EDPAAGNEGVAVDR-------------------------------------   859
SECA1_THE_fusca             786   EGIGLRAMAQRNPLIEYQREGYDMFQEMLEGIKEESIRFLFNVEVR----------VNQPQESQITAASAAATASAIPLVAPEAEKTEDKAEDAQEAEESAASAEAAESAKDTAQDKDAE   895
SECA2_MYC_vanbaalenii       783   AGIGMRALAGADPVVEYHREGHRMFVRMLEAVKEQSIRSLFDATTR----------MLAPIMPSA----ARSPGTPDPRWPF--------------------------------------   850
SECA_THE_maritima_          774   EAVQLRSYGQKDPIVEFKKETYYMFDEMMRRINDTIANYVLRVVKV-----------SEKDEKEAKE--ELGKIRLVHEEFNLVNRAMRR------------------------------   850
SECA2_GEO_kaustophilus      737   EGIHLRSYGQEDPYRAFEMDAYREFVALQQAIDAGICTTAMNYLKS-----------QFVIDDEAAA--AANKP----------------------------------------------   797
SECA2_GEO_thermodenitrifi   735   EGIGLRHYQQEDPIRLYQKEGFEMFKAMYEVIEKEISVHTARLLQS-----------LEQEEGQS-------------------------------------------------------   788
SECA2_BAC_anthracis         738   EGIGLRQYQQEDPTRLYQKEALDIFLYTYGNFEKEMCRYVARHLGV-----------PENVQ----------------------------------------------------------   788
SECA2_BAC_thuringiensis     738   EGIGLRQYQQEDPTRLYQKEALDIFLYTYGNFEKEMCRYVARHLGV-----------PENVQ----------------------------------------------------------   788
SECA2_BAC_cereus            738   EGIGLRQYQQEDPTRLYQKEALDIFLYTYGNFEKEMCRYVARHLGV-----------PENVQ----------------------------------------------------------   788
SECA2_LIS_welshimeri        726   EGIHLRAYGQQDPLVMYQKEGAQLFEKFQADYHFYFAHALLELDPD-----------GLVQG----------------------------------------------------------   776
SECA2_LIS_monocytogenes     726   EGIHLRAYGQQDPLVMYQKEGAQLFEKFQADYHFYFAHALLELDPD-----------GLIQG----------------------------------------------------------   776
SECA2_LIS_innocua           726   EGIHLRAYGQQDPLVMYQKEGAQLFEKFQADYHFYFAHALLELDPD-----------GLIQG----------------------------------------------------------   776
SECA2_ALK_metalliredigens   686   EGIHLVVIGKENPLHRFQSITIEAFNDMLDNIEKDIIETFIKANVT-----------KDGVDIEKEK--LKGPSSTWTYLISDSPNQFSRLP----------------------------   764
SECA2_LAC_johnsonii         731   INAQQWGRSGRPQDLLHQEKAFEAYKDFLDKITLSTFDNLLLSKIF-----------TNEKGQLVVV--FN-------------------------------------------------   788
SECA2_STA_haemolyticus      739   ASVNNRQNGQRNAIFEYHKVALETYEMMLINIKRATIRNLCLSILT-----------FDKDQDLVVH--FP-------------------------------------------------   796
SECA2_STA_aureus            739   ASVNQRQNGQRNAIFEYHRVALDSFEVMTRNIKKRMVKNICQSMIT-----------FDKEGMPVIH--FP-------------------------------------------------   796
SECA2_STA_epidermidis       739   ASVNNRQNGQRNVIFEYHKVALETYEYMSEDIKRKMVRNLCLSILA-----------FDKDGDMVIH--FP-------------------------------------------------   796
SECA2_STR_Agal              738   TVATARQTAQRNPVFEYHKEAYKSYNIMKKEIREQTFRNLLLSEVS-----------FNENGDLQIY--FI-------------------------------------------------   795
SECA2_STR_Gordon            736   SALSGFHTSNKKPIVEYYQEAYDGFEYMKERMKHQIVKNLLMSELA-----------LNPKGEVVMY--FP-------------------------------------------------   793
SECA2_STR_Pneum             733   MAIGGQSASQKNPIVEYYQEAYAGFEAMKEQIHADMVRNLLMGLVE-----------VTPKGEIVTH--FP-------------------------------------------------   790
SECA2_STR_Sanguin           736   TALSGQHFSMKNPLVEYYQEAYDGFEYMKERMKQQIVKNLLMSELA-----------LNPKGEVIMY--FP-------------------------------------------------   793
SECA2_COR_efficiens         675   ESIHLRAIARETPLDEYHRIAVREFKTLAQQAVDDAVETFRTVVID-----------DRGAHLEDAG--LARPSATWTYMVSD-------------------------------------   744
SECA2_MYC_ulcerans          719   ESIHLRALGRQNPLDEFHRMAVDAFASLAADAIEAAQQTFETANVL-----------EDEPGLDLSK--LARPTSTWTYMVND-------------------------------------   788
SECA2_MYC_paratuberculosi   690   ESIHLRALGRQNPLDEFHRLAVDAFASLAADAIEAAQQTFETANVL-----------EDEPGLDLSK--LARPTSTWTYMVND-------------------------------------   759
SECA2_MYC_tuberculosis      721   ESIHLRALGRQNPLDEFHRMAVDAFASLAADAIEAAQQTFETANVL-----------DHEPGLDLSK--LARPTSTWTYMVND-------------------------------------   790
SECA2_MYC_bovis             721   ESIHLRALGRQNPLDEFHRMAVDAFASLAADAIEAAQQTFETANVL-----------DHEPGLDLSK--LARPTSTWTYMVND-------------------------------------   790
SECA2_MYC_leprae            691   ESIHLRALGRQSPLDEFHRLAVNAFALLAADAIEAAQQTFETANIL-----------DGAPGLDLSK--LARPTSTWTYMVND-------------------------------------   760
SECA2_MYC_smegmatis         697   ESIHLRALGRQNPLDEFHRMAVDAFASLAADAIEAAQQTFETAESV-----------ADEPGVDLSK--LARPTSTWTYMVHD-------------------------------------   766
SECA2_THE_fusca             676   EGIHLRFLGRRDPLDEFNRDAVPAFKGFLDEARARAAEMFEKLEVV-----------DGRLDVAAAG--VKRPSTTWTYMVQD-------------------------------------   745
SECA2_COR_diphtheriae       676   ESIHLRTIARETPLDEYHRIAVREFKQLAQRAVDLAVETFRDVTID-----------QDGAHLADAG--LTRPSATWTYMVSD-------------------------------------   745
SECA2_COR_glutamicum        675   ESIHLRAIARETPLDEYHRIAVREFKDLAQRAVDDAVSTFKSVTID-----------HEGAHLDDEG--LARPSATWTYMVSD-------------------------------------   744
SECA2_COR_jeikeium          683   ESIHLRAIARESPIDEFHRMSIAAFGELAERAVNKARETFDEVEIT-----------SDGAQLGEMG--LHKPSATWTYMVND-------------------------------------   752
Consensus_aa:                     ptl.hR.htppsPl.b@ppbthp.F..h..php..hhp.h...............................................................................
Consensus_ss:                     hhhhhhhhhh  hhhhhhhhhhhhhhhhhhhhhhhhhhhhhh                      hhh  h
```

```
Conservation:
SECA1_GEO_thermodenitrifi   795  -------------------------VHPKE--DGEEPKKKPIR----KAVRVGRNDPCPCGS---------GKKYKHCCGRAV---------------  837
SECA1_GEO_kaustophilus      795  -------------------------VHPKE--DGEEPKRKPVR----KAVRVGRNDPCPCGS---------GKKYKHCCGRTV---------------  837
SECA1_LAC_johnsonii              --------------------------------------------------------------------------------------------------
SECA1_STR_parasanguinis     794  -------------------------STTAT--RNIAAQQQDIPA-DIDLSQVKRNDLCPCGS---------GKKFKNCHGRKF---------------  839
SECA1_STR_gordonii          794  -------------------------VTTAT--RNISAQESDLPA-DVDLAKVGRNELCPCGS---------GKKFKNCHGRR----------------  838
SECA1_STR_pneumoniae        792  -------------------------STTAT--RNIAAHQASMPE-DLDLSQIGRNELCPCGS---------GKKFKNCHGKRQ---------------  837
SECA1_STR_agalactiae        794  -------------------------TTTAE--QNISAQHVPMNNESPEYQGIKRNDKCPCGS---------GMKFKNCHGLR-CLQ------------  842
SECA1_LIS_monocytogenes     795  -------------------------INPAE--GKPEAKRQPIR----KDQHIGRNDPCPCGS---------GKKYKNCHGKEA---------------  837
SECA1_LIS_innocua           795  -------------------------INPAE--GKPEAKRQPVR----KDQHIGRNDPCPCGS---------GKKYKNCHGKEA---------------  837
SECA1_LIS_welshimeri_       795  -------------------------INPAE--GKPEAKRQPVR----KDQHIGRNDPCPCGS---------GKKYKNCHGKEA---------------  837
SECA_BAC_subtilis           797  -------------------------HQPQEGDDNKKAKKAPVR----KVVDIGRNAPCHCGS---------GKKYKNCCGRTE---------------  841
SECA1_BAC_anthracis         793  -------------------------VHPSS--DGEEAKKKPVV----KGDQVGRNDLCKCGS---------GKKYKNCCGIGK---------------  835
SECA1_BAC_thuringiensis     793  -------------------------VHPSS--DGEEAKKKPVV----KGDQVGRNDLCKCGS---------GKKYKNCCGIGK---------------  835
SECA1_BAC_cereus            793  -------------------------VHPSS--DGEEAKKKPVV----KGDQVGRNDLCKCGS---------GKKYKNCCGIGK---------------  835
SECA1_STA_epidermidis_      803  -------------------------VTAED--GKEKAKPQPIV----KGDQVGRNDPCPCGS---------GKKYKNCHGKA----------------  844
SECA1_STA_haemolyticus      803  -------------------------VSAED--GKEKAKKQPIV----KGDKVGRNDPCPCGS---------GKKYKNCHGKEE---------------  845
SECA1_STA_aureus            803  -------------------------VSAED--GKEKVKPKPIV----KGDQVGRNDDCPCGS---------GKKFKNCHGK-----------------  843
SECA1_ALK_metalliredigens   851  -------------------------IEASH--GDGNRKKAPVV----KEKEAGRNDPCPCGS---------GKKYKKCCGE-----------------  891
SECA1_PEP_difficile         851  -------------------------LSSPS--DGTLPTSKTVK----KDEKVGRNDLCPCGS---------GKKYKNCCGR-----------------  891
SECA2_PEP_difficile              --------------------------------------------------------------------------------------------------
SECA2_model_opened_conf          --------------------------------------------------------------------------------------------------
SECA_E_coli_                857  -------------------------LSHQD--DDSAAAAALAAQ--TGERKVGRNDPCPCGS---------GKKYKQCHGRLQ---------------  901
SECA1_MYC_vanbaalenii       877  -------VGGLRAKGIDDKAQPLTYTGPSE--DGG-------------VEVKRSGGGTPST----------GGTRKERREAA-RQQKT---GRHAKRR  938
SECA1_MYC_ulcerans          878  ------APSRLRAKGIEDESPALTYSGPSE--DGS-------------AQVQRNGGGAAKTPAG----VPAGRSRRERREAARRQGRGAKPPKSVKKR  950
SECA1_MYC_paratuberculosi   872  ---------QLRAKGIDNEAPAMTYSGPSE--DGS-------------AQVQRNGGDAKTPAG-----VPAGASRRERRAAARQQGRGAKPPKSVKKR  940
SECA1_MYC_tuberculosis      878  -------PSALRAKGVASESPALTYSGPAE--DGS-------------AQVQRNGGGAHKTPAG----VPAGASRRERREAARRQGRGAKPPKSVKKR  949
SECA1_MYC_bovis             878  -------PSALRAKGVASESPALTYSGPAE--DGS-------------AQVQRNGGGAHKTPAG----VPAGASRRERREAARRQGRGAKPPKSVKKR  949
SECA1_MYC_leprae            873  ----------RTKDIDNESTGLTYSGPSE--DGS-------------TQVQLNSGGGQKTPAG----IPVGASRRERREAARRRGRGAKPSRSVKKR  940
SECA1_MYC_smegmatis         880  --EAERPAPALRAKGIDNEAPPLTYTGPSE--DGT-------------AQVQRSGNGGRHAA-------PAGGSRRERREAARKQAKADRPAKSHRKG  953
SECA1_COR_diphtheriae            --------------------------------------------------------------------------------------------------
SECA1_COR_efficiens              --------------------------------------------------------------------------------------------------
SECA1_COR_glutamicum             --------------------------------------------------------------------------------------------------
SECA1_COR_jeikeium          860  -------------------------ATQTT--MGG---------------------------------------------------------------  867
SECA1_THE_fusca             896  SVAKKAQAVVPALGKEEKQPEKLQYSGPSE--GGG-------------VEKRTEDTGPDYANTPRNAPCPCGSGKKYKKCHG-APKSRV---------  968
SECA2_MYC_vanbaalenii            --------------------------------------------------------------------------------------------------
SECA_THE_maritima_          851  ------------------ATEKKKKDGLHS-----------------FGRIRVKR-------------------------------------------  871
SECA2_GEO_kaustophilus           --------------------------------------------------------------------------------------------------
SECA2_GEO_thermodenitrifi        --------------------------------------------------------------------------------------------------
SECA2_BAC_anthracis              --------------------------------------------------------------------------------------------------
SECA2_BAC_thuringiensis          --------------------------------------------------------------------------------------------------
SECA2_BAC_cereus                 --------------------------------------------------------------------------------------------------
SECA2_LIS_welshimeri             --------------------------------------------------------------------------------------------------
SECA2_LIS_monocytogenes          --------------------------------------------------------------------------------------------------
SECA2_LIS_innocua                --------------------------------------------------------------------------------------------------
SECA2_ALK_metalliredigens   765  ---------------FIVKGVLFSLRSISN-----------------GIVRKVQETINVVIQK-----------------------------------  795
SECA2_LAC_johnsonii              --------------------------------------------------------------------------------------------------
SECA2_STA_haemolyticus           --------------------------------------------------------------------------------------------------
SECA2_STA_aureus                 --------------------------------------------------------------------------------------------------
SECA2_STA_epidermidis            --------------------------------------------------------------------------------------------------
SECA2_STR_Agal                   --------------------------------------------------------------------------------------------------
SECA2_STR_Gordon                 --------------------------------------------------------------------------------------------------
SECA2_STR_Pneum                  --------------------------------------------------------------------------------------------------
SECA2_STR_Sanguin                --------------------------------------------------------------------------------------------------
SECA2_COR_efficiens         745  -------------------------------------------------------NPLAGS------------------GNS-VISGI---GNIFR--  763
SECA2_MYC_ulcerans          789  -------------------------------------------------------NPLSDD------------------TLS-TLSLP----GVFR--  806
SECA2_MYC_paratuberculosi   760  -------------------------------------------------------NPLSDD------------------TLS-TLSLP----GVFR--  777
SECA2_MYC_tuberculosis      791  -------------------------------------------------------NPLSDD------------------TLS-ALSLP----GVFR--  808
SECA2_MYC_bovis             791  -------------------------------------------------------NPLSDD------------------TLS-ALSLP----GVFR--  808
SECA2_MYC_leprae            761  -------------------------------------------------------APLSDD------------------TLS-PLSLP----GVFR--  778
SECA2_MYC_smegmatis         767  -------------------------------------------------------NPLADD------------------TMS-ALSLP----GVFR--  784
SECA2_THE_fusca             746  -------------------------------------------------------QPFSTD------------------LEN-IVGRV---KNLMGRD  766
SECA2_COR_diphtheriae       746  -------------------------------------------------------NPLSNN------------------NRS-VINGI---GSIFR--  764
SECA2_COR_glutamicum        745  -------------------------------------------------------NPLAGS------------------GNS-VISGI---GNIFR--  763
SECA2_COR_jeikeium          753  -------------------------------------------------------NPLSSS------------------GGS-VMGSI---VQMFR--  771
Consensus_aa:                    ..................................................................................................
Consensus_ss:
```

Figure S2, related to Figures 1, 2, 3 and 5: Structure-restrained sequence alignment of SecA1 and SecA2 sequences from 30 SecA2-encoding organisms. PROMALS3D alignment [2] of the 30 sequences encoding SecA1 (30 seq) and SecA2 (30 seq) was performed using default parameters, the coordinate file for CDSecA2 and the structure-based constraints from therein. So far, most of the SecA-protein interaction studies were based on the *T. maritima*, *E. coli* and *B. subtilis* SecAs. Thus, their sequences are added for orientation, but were not included in the Consurf analyses presented in main text (Figure 5). The highly conserved ATP binding site within Walker motifs is highlighted in red, the PPDX loop in cyan (coloured cyan in Figure 3A), the 2HF loop in yellow, and HWD domain in purple. The signal and nascent peptide binding sites (see Figures 3A, 3C and 5) involve residues present at the surface of the SecA molecule that make up or surround the groove. The ones that have been previously implicated in interaction with the signal peptide are highlighted in blue [3–7], and the ones implicated in the nascent protein binding in yellow [8–10]. The red frames represent the differences we observed when structurally aligning the CDSecA2 with *T. maritima* SecA, and refer to Figure 3B. The grey colouring represents the residues predicted to interact with the SecY channel [11–13]. The first line of each block shows conservation indices for positions with a conservation index above 4. Consensus predicted secondary structures (ss) are presented as symbols h for α-helix and b for β-strand. Conserved amino acids are shown in bold uppercase letters, aliphatic as *l*, aromatic as @, hydrophobic as *h*, alcohol as o, polar as p, tiny as t, small as s, bulky as b, positively charged as +, negatively charged as -, charged as c.

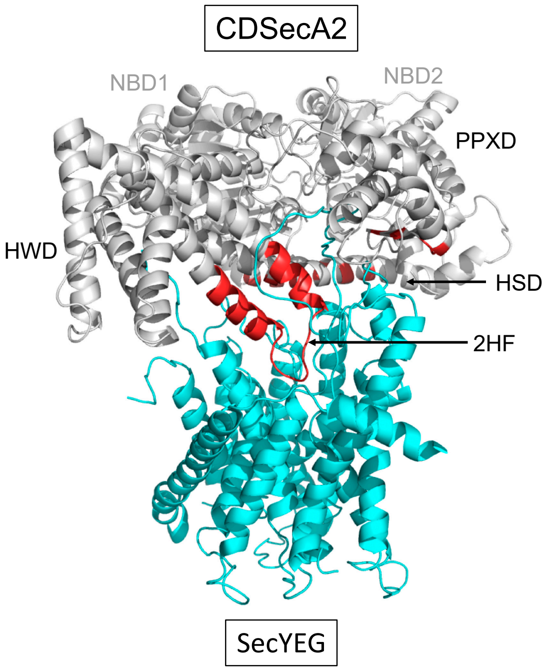

Figure S3, related to Figure 3, 4 and 5. Interface residues (red) of CDSecA2 (grey) that would contact the SecYEG (cyan), based on superposition with *T. maritima* SecA (PDB ID 3DIN; [11]).

SUPPLEMENTAL REFERENCES:

1. Schrödinger L. The PyMOL molecular graphics system, version 1.8. https://www.pymol.org/citing. 2015.
2. Pei J, Kim BH, Grishin N V. PROMALS3D: A tool for multiple protein sequence and structure alignments. Nucleic Acids Res. 2008.
3. Auclair SM, Moses JP, Musial-Siwek M, Kendall DA, Oliver DB, Mukerji I. Mapping of the Signal Peptide-Binding Domain of Escherichia coli SecA Using Förster Resonance Energy Transfer. Biochemistry. 2010;49:782–92. doi:10.1021/bi901446r.
4. Gelis I, Bonvin AMJJ, Keramisanou D, Koukaki M, Gouridis G, Karamanou S, et al. Structural Basis for Signal-Sequence Recognition by the Translocase Motor SecA as Determined by NMR. Cell. 2007.
5. Grady LM, Michtavy J, Oliver DB. Characterization of the Escherichia coli SecA signal peptide-binding site. J Bacteriol. 2012.
6. Musial-Siwek M, Rusch SL, Kendall DA. Probing the affinity of SecA for signal peptide in different environments. Biochemistry. 2005.
7. Kourtz L, Oliver D. Tyr-326 plays a critical role in controlling SecA-preprotein interaction. Mol Microbiol. 2000.

8. Zimmer J, Rapoport TA. Conformational Flexibility and Peptide Interaction of the Translocation ATPase SecA. J Mol Biol. 2009.
9. Erlandson KJ, Miller SBM, Nam Y, Osborne AR, Zimmer J, Rapoport TA. A role for the two-helix finger of the SecA ATPase in protein translocation. Nature. 2008;455:984–7. doi:10.1038/nature07439.
10. Bauer BW, Rapoport TA. Mapping polypeptide interactions of the SecA ATPase during translocation. Proc Natl Acad Sci. 2009;106:20800–5. doi:10.1073/pnas.0910550106.
11. Zimmer J, Nam Y, Rapoport TA. Structure of a complex of the ATPase SecA and the protein-translocation channel. Nature. 2008.
12. Swanson S, Ioerger TR, Rigel NW, Miller BK, Braunstein M, Sacchettini JC. Structural similarities and differences between two functionally distinct SecA proteins, Mycobacterium tuberculosis SecA1 and SecA2. J Bacteriol. 2016;198:720–30.
13. Das S, Oliver DB. Mapping of the SecA·SecY and SecA·SecG interfaces by site-directed in vivo photocross-linking. J Biol Chem. 2011.
